# In Silico RAPD Priming Sites in Expressed Sequences and iSCAR Markers for Oil Palm

**DOI:** 10.1155/2012/913709

**Published:** 2012-03-13

**Authors:** Balakrishnan Vasanthakumari Premkrishnan, Vadivel Arunachalam

**Affiliations:** ^1^Department of Computational Biology & Bioinformatics, North Campus-Kariavattom, University of Kerala, Thiruvananthapuram, Kerala 695581, India; ^2^Horticulture Section, ICAR Research Complex for Goa, Ela Old Goa 403402, India

## Abstract

RAPD is a simple dominant marker system widely used in biology. Effectiveness of RAPD can be improved by selecting and redesigning primers whose priming sites occur in target sequence(s) of gene or organism at optimum distance. We developed software that uses sequences of random decamer primers and nucleotide sequence(s) as two input files. It locates the priming sites in input sequences and generates output files listing frequency and distance between priming sites. When the priming sites of a single primer occur more than once in a sequence with a distance of 200 to 2000 bp, the software also designs pairs of iSCAR primers. An input of 387 RAPD primers and 42,432 expressed sequences of oil palm are used as test. Wet-lab PCR results from a publication that used the same set of primers were compared with software output on priming sites. In the test sequences of oil palm covering 1.4% of genome, we found that at least 60% the primers chosen using software are sure of giving PCR amplification. We designed 641 iSCAR primers suitable for amplification of oil palm DNA. The software successfully predicted 92% (67 out of 73) of published polymorphic RAPD primers in oil palm.

## 1. Introduction

The secret of differences between individual organism lies in their genetic material, called deoxyribonucleic acid (DNA). A genetic marker can be defined in one of the following ways: (a) a chromosomal landmark or allele that allows for the tracing of a specific region of DNA, (b) a specific piece of DNA with a known position on the genome, or (c) a gene whose phenotypic expression is usually easily discerned, used to identify an individual or a cell that carries it, or as a probe to mark a nucleus, chromosomes, or locus [1]. Genetic markers may not have a biological function, and they are inherited from one generation to next.

Random amplified polymorphic (RAPD) DNA markers were introduced by Williams et al. [2] in 1990. RAPD markers can be implemented more rapidly and inexpensively than other type of markers. Prior knowledge of the DNA sequence for the targeted gene is not required, as the primers will bind somewhere in the sequence although exact location is unknown. RAPD primers are of decamer (10 base pairs) size and are randomly generated. The success of Polymerase Chain Reaction (PCR) is highly dependent on these short arbitrary oligonucleotides that hybridize onto the complementary DNA fragments. These short oligonucleotides function in pairs (one forward and one reverse primer) and are used to amplify [3] a set of DNA fragments. When choosing an arbitrary primer for a reaction, two basic criteria [4] must be met: a minimum of 40% GC content (50%–80% GC content is generally used) and the absence of a palindromic sequence (a base sequence that reads exactly the same from right to left as from left to right). RAPD primers can be purchased in sets or individually using sequences from different sources (e.g., Operon Biotechnologies— http://www.operon.com/). RAPD markers are widely used for finding genetic diversity [5–8] and genetic relationships [9] in plants and animals. Recent studies have proved that they can be used to predict phylogenetic relationship analysis [10, 11] and genome specificity of catfishes [10]. These markers can also be used in identifying genetic variation between plant species [12] and also in animals [13].

A sequence characterized amplified region (SCAR) marker is a genomic DNA fragment that is identified by PCR amplification using a pair of specific oligonucleotide primers [14, 15]. SCARs are derived by cloning and sequencing the unique bands whose presence or absence is diagnostic for specific purposes [4]. After getting the sequence of the amplified band analyzed, the same is analyzed and subjected to primer design. Forward and reverse primers each of approximately 20 bp size are designed in the sequence now known flanking the previous RAPD region(s). SCARs are advantages over RAPD markers as they detect only a single locus, their amplification is less sensitive to reaction conditions, and they can potentially be converted into codominant markers [14]. SCAR marker can also be used to compare genetic relationships among different plant species [16].

Expressed sequence tags (ESTs) are used to reveal gene expression patterns, gene regulation, and sequence diversity between subspecies of plants on a large scale. They can also be used for the discovery of important tissue-specific genes [17]. ESTs proved that they can be used successfully for the comparative genomics studies [18] between Oil palm and other monocotyledonous and dicotyledonous plants. Comparative studies were done to explore the possibilities of both RAPD and EST in which they have proved that ESTs can show a high coincident bias pattern with that of the whole genomic sequence and therefore be used to assess efficiencies of primers for species [19] whose genomic sequence data are currently unknown.

Oil palm (*Elaeis guineensis *Jacq.) fruit is a drupe with a thick fleshy mesocarp. Mesocarp tissue is rich in oil (80% dry weight basis) content. Oil palm is the highest oil-yielding crop of the world [20]. Shell thickness is the important trait in oil palm which differs between the dura and pisifera, the two common fruit forms of oil palm. Dura is a form with thick shelled kernel whereas pisifera is a thin shelled kernel type. Tenera is a high yielding hybrid between dura (*Sh* ^+^ *Sh* ^+^) and pisifera (*Sh* ^−^ *Sh* ^−^) forms of oil palms differing in shell thickness (*Sh* ^+^) trait. A single locus with two alleles in a codominant fashion controls the shell thickness in tenera form (*Sh* ^+^ *Sh* ^−^) [21, 22] of oil palm. Fruits of the pisifera form in oil palm are associated with failure of lignin and fiber synthesis around the shell. Pisifera palms are usually female sterile and are difficult to propagate by seeds except by embryo rescue techniques.

The endocarp cells of oil palm fruit get lignified to form a hard shell while the mesocarp tissue remains fibrous during the formation of shell formation. A transition zone made up of fibrous units was also visible beneath the shell. Enzymes such as Phenylalanine ammonia-lyase (PAL), cinnamyl alcohol-NADPH-dehydrogenase (CAD), and peroxidase (POD) played important role in lignin synthesis [23]. Quantification of activity of these lignin biosynthetic enzymes has potential to discriminate the seedlings as dura, pisifera, and tenera [3, 23]. Our interest in this study is to look for RAPD priming sites in the expressed sequences of oil palm and compare them with published RAPD markers in oil palm. Three important RAPD primers (OPR-11, OPT-19, and OPY-20) are closely related to shell thickness of oil palm [17]. Hence, we intend to locate priming sites of OPR-11, OPT-19, and OPY-20 in expressed sequences of oil palm mesocarp tissues. We also indent to design *insilico* sequence characterized amplified region (iSCAR) primers for shell thickness.

## 2. Materials and Methods

An in-house Perl script was developed to predict the number of priming sites and other relevant details. The software currently works on Windows (98 or above version), DOS, and Linux operating systems. The Perl script (tool) was written to (a) search for the priming sites of the given primer in all the target EST sequences, (b) calculate the priming sites of each sequences, (c) measure the distance between the priming sites, (d) calculate the base composition of the sequences, and (e) convert best amplifiable primers into pair of iSCAR primers.

The software was developed using the scripting language perl, which has powerful “string processing capabilities” and pattern match abilities. Basically the software is a pattern matching tool and can search for a given set of primer patterns in a target sequence set and records the details of each matching pattern found. This program takes two input files (text files and/or fasta files). One file should contain list and sequences of RAPD or any decamer primers, and other file should contain the target nucleotide sequence(s). Software reads the primer sequences one by one from the given RAPD primer file and will try to find the presence of the primer in the second target sequence file. If there is no matching of the primer, the program will take next primer from the file and continues with match finding process. Whenever a match is found, between the primer sequence and the target sequence, the software will record the details in the output file. If there is more than one matching for a specific primer in the same target sequence, the program will additionally create a separate file for recording such occurrences. The program will automatically search for matches in the target sequence by allowing a mismatch of 0–3 bases out of the 10 bases of the decamer primer. Priming site details output file will contain primers which have more than one matching site in the target sequence and gives the start and end positions of each matching priming site. Based on the distance between the priming sites, the anticipated product size for each primer is calculated by the software. Primers which are having a distance between priming sites in the specified range (200–2000 base pairs) are chosen for design for iSCAR primer pairs. iSCAR primers are designed by selecting an additional 10 bases from the target sequence closer to the priming site of the RPAD primer in both directions (forward and reverse). Software also considers the complementarity of each primer sequence in the given set and performs all the above steps for each of them. So the output of the program will create two sets of files, one for original RAPD priming sites and next for the complementary priming sites. In addition, the software also generates a base composition file that contains the details regarding the counts of each bases, GC content percentage, and so forth.

We have retrieved 42,432 EST sequences of 27.4 Mbp size from GenBank EST sequence database as available on August 01, 2011. About 420 deca-nucleotide primers were obtained (http://www.operon.com/) from Operon Technologies (CA, USA). A set of primer series each with 20 individual primers (OPA, OPB, OPC, OPD, OPE, OPF, OPG, OPI, OPJ, OPK, OPL, OPM, OPN, OPP, OPQ, OPR, OPS, OPT, OPX, OPY, and OPZ) were used in the study.

The published work of Rival et al. [24] on the use of RAPD markers on oil palm is used as known wet-lab PCR-validated data set and was used for the comparison of the software results. Primers giving exploitable amplification products and those that showed interclonal polymorphism are listed in the work [24]. Authors of the [24] study used a series of 387 decamer oligonucleotides, purchased from Operon Technologies, to investigate the genetic fidelity of somatic embryogenesis-derived regenerants of oil palm. The same set of primer sequences was used as input file in our software. Results of PCR validation of number of RAPD markers in oil palm from the published results [24] and priming sites of these primers using the software were compared and tabulated. About 73 RAPD primers displaying interclonal polymorphism in oil palm based on [24] the published work of Rival et al., (1999) swere chosen as input file. Priming sites of these 73 RAPD primers were searched in the expressed sequences of oil palm using our software.

## 3. Results and Discussion

In our study, a total of 400 primers (each primer from the given 20 primer series, excluding OPT series) were used as input. But authors of [24] used 387 primers from all the primer series. They have excluded some of the primers from the original RAPD kit. Of the 387 primers they [24] have used, 258 (67%) were successfully amplified in oil palm DNA with consistently reproducible banding. The size of the oil palm genome is approximately 1950 Mbp, and the sequences used in the current study of software by us covers only 27.40 Mbp (1.4% of genome). We had used only expressed sequences of the species that hence excludes the priming sites in introns and other noncoding regions of the genome. Our results show a 68% correspondence between published results and the software output on priming sites. Priming sites of 165 primers of our software output corresponded with the total 258 PCR-validated amplified [24] primers (64%). Priming sites of the remaining 93 primers are present in the rest of the genome that hence gave amplicons in PCR results of [24]. The priming sites of these 93 primers are probably absent in the expressed sequences used as input for our software test.

Although we had used a low proportion (1.4% of the genome) covering only the expressed region of genome, we found >60% of the primers which can successfully give amplification of genomic DNA because the numbers of priming sites of a RAPD primer can vary many fold (1 to 465) in genomic sequence of *Arabidopsis thaliana* [19] of size 120 Mbp. The use of software-based primer selection can reduce the amount of RAPD primers required for research by 50% [19]. In the test study on EST sequences of oil palm with 1.4% of genome data, we found that at least 60% the primers chosen using software are sure of giving PCR amplification. Hence the software has potential especially to reduce the number of primers needed for research on species whose sequence information available in public domain is limited.

Of these, 73 primers (19%) were able to distinguish polymorphism between clones by the authors [24]. Priming sites of these 73 RAPD primers were searched in the expressed sequences of oil palm using our software. We found priming sites of 67 RAPD primers occurring twice or more in the sequences analyzed. Out of these 73 polymorphic RAPD primers, priming sites of only six of them were absent in the sequences under study. Hence our software is capable of predicting 92% of the successful polymorphic RAPD primers in the case study of oil palm.

The publication [24] used whole genome sequence for the analysis but our tool used only the EST sequences. Out of 258 amplified primers present reported by authors of [24], priming sites of 165 were found in ESTs based on our study using the software results. Summary of comparative details of the results between publication and tool are given in Table 1.

 Information of RAPD priming sites, anticipated product size after PCR (See supplementary Table S1 available online at doi: 10.1155/2012/913709) and pairs of iSCAR primer sequences (Table S2) are provided as supplementary files. Primer bias pattern between the genomic sequence and uni-EST sequences [19] showed a strong coincidence pattern with a correlation coefficient of *r* = 0.94 by Li et al., (2006) [19]. Three RAPD primers (OPR-11, OPT-19, and OPY-20), associated with the shell thickness of oil palms, were also included in the analysis. Priming sites of these three sequences were found in some of the EST sequences. But the product size obtained was comparatively small hence iSCAR primers could not be designed.  Table 2 displays the number of priming sites for each primer in each of the tissue-specific libraries of oil palm. Priming sites in the library of mesocarp tissues are important as the shell is present in the mesocarp tissue. The software was successful in converting the predicted RAPD primers, which was having two or more priming sites, into iSCAR primers (Supplementary Table S2). The letter “i” represents that the output generated is an in silico result file. The iSCAR file contains (a) name of the primer, (b) identification number of the sequence in which the primer has annealed, (c) SCAR primer sequence in forward direction, and (d) SCAR primer sequence in the reverse direction.

Success of RAPD primers depends on the number and distance between priming sites in the target genome sequence. We need an in silico tool which can predict the priming sites of a given set of RAPD primers within a target sequences which can save time and money in use of selected primers whose priming sites are at amplifiable distances in the genome. “eRAPD” software was developed for selecting efficient primers based on the primer bias [19] in the genomic sequence. But eRAPD [19] software does not have the option to design the iSCAR primers. RAPD primers whose priming sites occur only once in a sequence are less likely to give amplifiable bands. A RAPD primer having two or more priming sites at distance 200 bp to 2000 bp is preferred from the predicted result file. These primers are given as a separate output file. Our software is a user friendly tool for molecular biology researchers to shortlist the probable successful RAPD primers and design-suitable iSCAR primer pairs. RAPD primers are not specific to reaction agents, laboratory conditions, and hence sometimes they are not repeatable. Conversion of RAPD markers into sequenced characterized amplified region (SCAR) markers can overcome the above limitations. Sequencing of RAPD bands and developing SCAR markers are another development which makes RAPD markers into a reliable and repeatable marker system [25]. Interested researchers can contact author by email (vadivelarunachalam@yahoo.com) and get the services of the software free of cost for academic use and on charge for commercial use.

## 4. Conclusions

RAPD markers are very easy to use and cheaper than other type of genetic markers because of three main reasons: (a) they are only 10 base pairs in length, (b) can be chosen randomly, and (c) can be used with unknown target sequences. We developed software which selects the primers whose priming sites are available in the sequence and designs iSCAR primers. Hence it can boost the effectiveness of the RAPD marker system for reproducibility. We tested the software for the prediction of RAPD primers in the target sequence and for conversion to iSCAR markers in oil palm. A set of shortlisted RAPD (Supplementary table S1) and 641 iSCAR primers (Supplementary table S2) suitable for amplification of oil palm DNA are identified by the study. In the test sequences of oil palm covering 1.4% of genome, we found at least that 60% the primers chosen using software are sure of giving PCR amplification. The software was successful in locating 92% (67 out of 73) published polymorphic RAPD primers in oil palm. Software-aided selection of primers has potential to choose the most probable successful polymorphic primers especially on species with limited sequence information. 

## Supplementary Material

Details of the priming sites of RAPD primers in oil palm expressed sequnecs found by the software along with the anticipated product size, 2. List of in silico Sequence characterised amplified region primer pairs for oil palm.

## Figures and Tables

**Table 1:** Summary of results between output of software and published RAPD markers.

Primers used for the study	Amplified primers given [24] Rival et al., 1998	Priming sites of primers identified by the software	Amplified primers, present in publication and priming sites identified by software
400	258 (64.5%)	271 (67.75%)	165

**Table 2:** Number of priming sites of three RAPD primers associated with shell thickness in expressed sequences of oil palm.

Primer	Total number of priming sites	Number of priming sites in mesocarp tissue library	Number of priming sites in inflorescence tissue library	Number of priming sites in other tissues
OPR-11	89	5	6	78
OPT-19	76	6	2	66
OPY-20	258	49	11	198
