# Supplementary material for: In Silico RAPD Priming Sites in Expressed Sequences and iSCAR Markers for Oil Palm
Source: Comp Funct Genomics. 2012 Mar 13;2012:913709. doi: 10.1155/2012/913709 (PMC3313062; doi:10.1155/2012/913709)
Supplement: Supplementary file 1 — Details of the priming sites of RAPD primers in oil palm expressed sequnecs found by the software along with the anticipated product size, 2. List of in silico Sequence characterised amplified region primer pairs for oil palm. [file 913709.f1.pdf]

Supplementary table 1. **Comparison of the results of the tool and published results**

List of Primers Used	Primer Sequence	Amplified Primers		Product Size (predicted by the Tool)
		Identified In Publication	Identified by Tool	
OPA-01	CAGGCCCTTC	OPA-01		
OPA-02	TGCCGAGCTG	OPA-02	OPA-02	
OPA-03	AGTCAGCCAC		OPA-03	236
OPA-04	AATCGGGCTG	OPA-04		
OPA-05	AGGGGTCTTG		OPA-05	394
OPA-06	GGTCCCTGAC	OPA-06		
OPA-07	GAAACGGGTG	OPA-07		
OPA-08	GTGACGTAGG	OPA-08	OPA-08	1457, 319
OPA-09	GGGTAACGCC	OPA-09	OPA-09	
OPA-10	GTGATCGCAG	OPA-10	OPA-10	697, 260, 887, 703, 220
OPA-11	CAATCGCCGT	OPA-11		
OPA-12	TCGGCGATAG	OPA-12	OPA-12	
OPA-13	CAGCACCCAC	OPA-13	OPA-13	327, 423, 435
OPA-14	TCTGTGCTGG	OPA-14	OPA-14	266, 301
OPA-15	TTCCGAACCC	OPA-15		
OPA-16	AGCCAGCGAA	OPA-16	OPA-16	
OPA-17	GACCGCTTGT	OPA-17	OPA-17	
OPA-18	AGGTGACCGT	OPA-18		
OPA-19	CAAACGTCGG	OPA-19		
OPA-20	GTTGCGATCC	OPA-20		
OPB-01	GTTTCGCTCC	OPB-01		
OPB-02	TGATCCCTGG	OPB-02		
OPB-03	CATCCCCCTG	OPB-03	OPB-03	650, 241, 234, 463, 417, 393, 235, 493
OPB-04	GGACTGGAGT		OPB-04	209, 326
OPB-05	TGCGCCCTTC	OPB-05		
OPB-06	TGCTCTGCCC	OPB-06	OPB-06	457, 245, 253, 1195, 282, 406, 211
OPB-07	GGTGACGCAG	OPB-07	OPB-07	
OPB-08	GTCCACACGG	OPB-08	OPB-08	419, 600
OPB-09	TGGGGGACTC	OPB-09		
OPB-10	CTGCTGGGAC	OPB-10	OPB-10	200, 486
OPB-11	GTAGACCCGT	OPB-11		
OPB-12	CCTTGACGCA	OPB-12		

OPB-13	TTCCCCCGCT	OPB-13	OPB-13	356, 237, 453, 235
OPB-14	TCCGCTCTGG	OPB-14	OPB-14	336
OPB-15	GGAGGGTGTT	OPB-15	OPB-15	241, 493, 420, 211
OPB-16	TTTGCCCGGA	OPB-16	OPB-16	235
OPB-17	AGGGAACGAG	OPB-17	OPB-17	303
OPB-18	CCACAGCAGT	OPB-18	OPB-18	306
OPB-19	ACCCCCGAAG	OPB-19		
OPB-20	GGACCCTTAC	OPB-20		
OPC-01	TTCGAGCCAG	OPC-01	OPC-01	537
OPC-02	GTGAGGCGTC		OPC-02	359
OPC-03	GGGGGTCTTT	OPC-03	OPC-03	
OPC-04	CCGCATCTAC	OPC-04	OPC-04	202
OPC-05	GATGACCGCC			
OPC-06	GAACGGACTC	OPC-06	OPC-06	260
OPC-07	GTCCCGACGA	OPC-07		
OPC-08	TGGACCGGTG	OPC-08	OPC-08	
OPC-09	CTCACCGTCC	OPC-09	OPC-09	
OPC-10	TGTCTGGGTG	OPC-10	OPC-10	402
OPC-11	AAAGCTGCGG	OPC-11	OPC-11	521
OPC-12	TGTCATCCCC		OPC-12	
OPC-13	AAGCCTCGTC			
OPC-14	TGCGTGCTTG	OPC-14	OPC-14	661
OPC-15	GACGGATCAG		OPC-15	307, 300
OPC-16	CACACTCCAG	OPC-16	OPC-16	559, 741, 715, 248, 1617
OPC-17	TTCCCCCCAG	OPC-17	OPC-17	384, 387, 239, 263, 341, 331, 281, 200, 353, 692, 376, 338, 433, 399, 264, 599, 246, 240, 272, 203, 298, 418, 321, 242,
OPC-18	TGAGTGGGTG		OPC-18	
OPC-19	GTTGCCAGCC		OPC-19	403
OPC-20	ACTTCGCCAC		OPC-20	
OPD-01	ACCGCGAAGG	OPD-01	OPD-01	
OPD-02	GGACCCAACC		OPD-02	283, 534
OPD-03	GTCGCCGTCA	OPD-03	OPD-03	267
OPD-04	TCTGGTGAGG	OPD-04	OPD-04	429, 235, 430

OPD-05	TGAGCGGACA	OPD-05		
OPD-06	ACCTGAACGG	OPD-06	OPD-06	
OPD-07	TTGGCACGGG	OPD-07		
OPD-08	GTGTGCCCCA		OPD-08	
OPD-09	CTCTGGAGAC	OPD-09	OPD-09	471, 299
OPD-10	GGTCTACACC	OPD-10	OPD-10	
OPD-11	AGCGCCATTG	OPD-11		
OPD-12	CACCGTATCC	OPD-12	OPD-12	
OPD-13	GGGGTGACGA			
OPD-14	CTTCCCCAAG		OPD-14	468, 245, 258
OPD-15	CATCCGTGCT	OPD-15	OPD-15	408
OPD-16	AGGGCGTAAG	OPD-16		
OPD-17	TTTCCCACGG		OPD-17	269, 546
OPD-18	GAGAGCCAAC		OPD-18	
OPD-19	CTGGGGACTT			
OPD-20	ACCCGGTCAC	OPD-20		
OPE-01	CCCAAGGTCC	OPE-01	OPE-01	
OPE-02	GGTGCGGGAA	OPE-02	OPE-02	
OPE-03	CCAGATGCAC	OPE-03	OPE-03	404
OPE-04	GTGACATGCC	OPE-04	OPE-04	252, 1128
OPE-05	TCAGGGAGGT	OPE-05		
OPE-06	AAGACCCCTC	OPE-06	OPE-06	
OPE-07	AGATGCAGCC		OPE-07	403, 371, 206, 241, 597, 314, 442, 386, 469, 255, 781,
OPE-08	TCACCACGGT	OPE-08		
OPE-09	CTTCACCCGA	OPE-09	OPE-09	350, 355
OPE-10	CACCAGGTGA	OPE-10	OPE-10	315, 397
OPE-11	GAGTCTCAGG	OPE-11	OPE-11	
OPE-12	TTATCGCCCC	OPE-12		
OPE-13	CCCGATTCGG	OPE-13	OPE-13	663
OPE-14	TGCGGCTGAG	OPE-14	OPE-14	587
OPE-15	ACGCACAACC	OPE-15	OPE-15	231, 291, 1419
OPE-16	GGTGACTGTG	OPE-16		
OPE-17	CTACTGCCGT	OPE-17	OPE-17	1067
OPE-18	GGACTGCAGA	OPE-18	OPE-18	437, 440, 436, 385
OPE-19	ACGGCGTATG	OPE-19		
OPE-20	AACGGTGACC	OPE-20	OPE-20	242

OPF-01	ACGGATCCTG			
OPF-02	GAGGATCCCT		OPF-02	
OPF-03	CCTGATCACC		OPF-03	482, 310, 463, 224, 235
OPF-04	GGTGATCAGG	OPF-04	OPF-04	271, 434, 508
OPF-05	CCGAATTCCC	OPF-05	OPF-05	530, 393
OPF-06	GGGAATTCGG	OPF-06	OPF-06	237, 351, 235, 451
OPF-07	CCGATATCCC	OPF-07	OPF-07	
OPF-08	GGGATATCGG	OPF-08	OPF-08	
OPF-09	CCAAGCTTCC	OPF-09	OPF-09	319, 379
OPF-10	GGAAGCTTGG	OPF-10	OPF-10	510, 607, 658
OPF-11	TTGGTACCCC	OPF-11	OPF-11	1307
OPF-12	ACGGTACCAG	OPF-12		
OPF-13	GGCTGCAGAA	OPF-13	OPF-13	340, 305, 223, 430
OPF-14	TGCTGCAGGT	OPF-14	OPF-14	310, 280, 346, 401, 212, 319, 201, 321, 634
OPF-15	CCAGTACTCC	OPF-15	OPF-15	
OPF-16	GGAGTACTGG	OPF-16	OPF-16	316
OPF-17	AACCCGGGAA	OPF-17	OPF-17	237, 334, 498, 310, 282, 230
OPF-18	TTCCCGGGTT	OPF-18	OPF-18	
OPF-19	CCTCTAGACC	OPF-19	OPF-19	612
OPF-20	GGTCTAGAGG	OPF-20		
OPG-01	CTACGGAGGA			
OPG-02	GGCACTGAGG		OPG-02	223
OPG-03	GAGCCCTCCA		OPG-03	
OPG-04	AGCGTGTCTG	OPG-04	OPG-04	353
OPG-05	CTGAGACGGA	OPG-05		
OPG-06	GTGCCTAACC	OPG-06	OPG-06	403, 369
OPG-07	GAACCTGCGG			
OPG-08	TCACGTCCAC	OPG-08		
OPG-09	CTGACGTCAC	OPG-09		
OPG-10	AGGGCCGTCT	OPG-10		
OPG-11	TGCCCGTCGT	OPG-11	OPG-11	307
OPG-12	CAGCTCACGA	OPG-12	OPG-12	
OPG-13	CTCTCCGCCA	OPG-13	OPG-13	515
OPG-14	GGATGAGACC	OPG-14	OPG-14	694, 1062, 530
OPG-15	ACTGGGACTC	OPG-15		

OPG-16	AGCGTCCTCC	OPG-16		
OPG-17	ACGACCGACA			
OPG-18	GGCTCATGTG	OPG-18	OPG-18	408, 264, 407, 410
OPG-19	GTCAGGGCAA	OPG-19		
OPG-20	TCTCCCTCAG	OPG-20	OPG-20	333, 563, 675, 328, 330, 374, 497, 230, 420, 327, 1664, 409, 284, 508
OPI-01	ACCTGGACAC			
OPI-02	GGAGGAGAGG		OPI-02	204, 241, 460, 264, 222, 297, 587, 240, 280, 471, 381, 268, 220, 277, 584, 239, 287, 208, 266, 205, 212, 214, 235, 238, 476, 211, 367, 408, 431, 296, 311, 768
OPI-03	CAGAAGCCCA		OPI-03	315, 266
OPI-04	CCGCCTAGTC			
OPI-05	TGTTCCACGG	OPI-05		
OPI-06	AAGGCGGCAG		OPI-06	504
OPI-07	CAGCGACAAG		OPI-07	856, 394
OPI-08	TTTGCCCGGT	OPI-08	OPI-08	235
OPI-09	TGGAGAGCAG		OPI-09	291, 283, 433
OPI-10	ACAACGCGAG	OPI-10	OPI-10	648
OPI-11	ACATGCCGTG		OPI-11	
OPI-12	AGAGGGCACA		OPI-12	274, 463, 459
OPI-13	CTGGGGCTGA		OPI-13	324
OPI-14	TGACGGCGGT		OPI-14	206
OPI-15	TCATCCGAGG	OPI-15		
OPI-16	TCTCCGCCCT		OPI-16	271, 643, 605, 204, 298, 406, 211, 351, 574
OPI-17	GGTGGTGATG		OPI-17	356, 367, 1522, 286, 289, 228, 433, 250, 287, 325
OPI-18	TGCCCAGCCT			
OPI-19	AATGCGGGAG	OPI-19		
OPI-20	AAAGTGCGGG			

OPJ-01	CCCGGCATAA			
OPJ-02	CCCGTTGGGA			
OPJ-03	TCTCCGCTTG	OPJ-03	OPJ-03	
OPJ-04	CCGAACACGG	OPJ-04	OPJ-04	
OPJ-05	CTCCATGGGG		OPJ-05	295, 762
OPJ-06	TCGTTCCGCA	OPJ-06		
OPJ-07	CCTCTCGACA		OPJ-07	496
OPJ-08	CATACCGTGG	OPJ-08	OPJ-08	1133
OPJ-09	TGAGCCTCAC		OPJ-09	235
OPJ-10	AAGCCCGAGG		OPJ-10	
OPJ-11	ACTCCTGCGA		OPJ-11	326, 1100
OPJ-12	GTCCCGTGGT	OPJ-12		
OPJ-13	CCACACTACC		OPJ-13	1400
OPJ-14	CACCCGGATG		OPJ-14	
OPJ-15	TGTAGCAGGG	OPJ-15	OPJ-15	228, 340
OPJ-16	CTGCTTAGGG		OPJ-16	
OPJ-17	ACGCCAGTTC			
OPJ-18	TGGTCGCAGA		OPJ-18	463, 235
OPJ-19	GGACACCACT		OPJ-19	
OPJ-20	AAGCGGCCTC			
OPK-01	CATTCGAGCC	OPK-01		
OPK-02	GTCTCCGCAA		OPK-02	620, 235
OPK-03	CCAGCTTAGG	OPK-03	OPK-03	340
OPK-04	CCGCCCAAAC		OPK-04	241, 297
OPK-05	TCTGTCGAGG	OPK-05		
OPK-06	CACCTTTCCC	OPK-06	OPK-06	
OPK-07	AGCGAGCAAG		OPK-07	
OPK-08	GAACACTGGG	OPK-08		
OPK-09	CCCTACCGAC	OPK-09	OPK-09	
OPK-10	GTGCAACGTG	OPK-10		
OPK-11	AATGCCCCAG		OPK-11	
OPK-12	TGGCCCTCAC			
OPK-13	GGTTGTACCC	OPK-13		
OPK-14	CCCGCTACAC	OPK-14		
OPK-15	CTCCTGCCAA		OPK-15	249, 313
OPK-16	GAGCGTCGAA	OPK-16	OPK-16	519, 470
OPK-17	CCCAGCTGTG	OPK-17	OPK-17	446, 369, 491
OPK-18	CCTAGTCGAG	OPK-18		
OPK-19	CACAGGCGGA		OPK-19	251

OPK-20	GTGTCGCGAG	OPK-20	OPK-20	558
OPL-01	GGCATGACCT			
OPL-02	TGGGCGTCAA		OPL-02	
OPL-03	CCAGCAGCTT		OPL-03	234, 347, 268, 235, 609
OPL-04	GACTGCACAC	OPL-04	OPL-04	943, 1390, 616
OPL-05	ACGCAGGCAC		OPL-05	
OPL-06	GAGGGAAGAG	OPL-06	OPL-06	594, 223
OPL-07	AGGCGGGAAC		OPL-07	372
OPL-08	AGCAGGTGGA		OPL-08	245
OPL-09	TGCGAGAGTC	OPL-09	OPL-09	598, 247
OPL-10	TGGGAGATGG		OPL-10	261, 570, 508
OPL-11	ACGATGAGCC		OPL-11	1902, 938, 1908, 704
OPL-12	GGGCGGTACT	OPL-12		
OPL-13	ACCGCCTGCT	OPL-13	OPL-13	757
OPL-14	GTGACAGGCT			
OPL-15	AAGAGAGGGG		OPL-15	427, 567, 342, 500, 266, 343
OPL-16	AGGTTGCAGG	OPL-16	OPL-16	643
OPL-17	AGCCTGAGCC	OPL-17		
OPL-18	ACCACCCACC	OPL-18	OPL-18	205, 312, 295, 509, 270, 223
OPL-19	GAGTGGTGAC		OPL-19	890, 870, 504, 223
OPL-20	TGGTGGACCA	OPL-20	OPL-20	302, 235
OPM-01	GTTGGTGGCT		OPM-01	466, 304, 532, 202
OPM-02	ACAACGCCTC	OPM-02	OPM-02	244
OPM-03	GGGGGATGAG	OPM-03	OPM-03	277
OPM-04	GGCGGTTGTC		OPM-04	
OPM-05	GGGAACGTGT		OPM-05	303, 222
OPM-06	CTGGGCAACT	OPM-06		
OPM-07	CCGTGACTCA	OPM-07		
OPM-08	TCTGTTCCCC		OPM-08	
OPM-09	GTCTTGCGGA	OPM-09	OPM-09	492
OPM-10	TCTGGCGCAC	OPM-10		
OPM-11	GTCCACTGTG	OPM-11	OPM-11	419
OPM-12	GGGACGTTGG			
OPM-13	GGTGGTCAAG	OPM-13	OPM-13	289

OPM-14	AGGGTCGTTC	OPM-14		
OPM-15	GACCTACCAC	OPM-15		
OPM-16	GTAACCAGCC	OPM-16	OPM-16	
OPM-17	TCAGTCCGGG			
OPM-18	CACCATCCGT		OPM-18	272, 268, 336
OPM-19	CCTTCAGGCA	OPM-19	OPM-19	
OPM-20	AGGTCTTGGG		OPM-20	245
OPN-01	CTCACGTTGG	OPN-01	OPN-01	
OPN-02	ACCAGGGGCA	OPN-02	OPN-02	
OPN-03	GGTACTCCCC	OPN-03		
OPN-04	GACCGACCCA		OPN-04	
OPN-05	ACTGAACGCC		OPN-05	
OPN-06	GAGACGCACA		OPN-06	957, 594, 236
OPN-07	CAGCCCAGAG	OPN-07	OPN-07	
OPN-08	ACCTCAGCTC		OPN-08	252, 902, 330, 366, 250, 262
OPN-09	TGCCGGCTTG		OPN-09	
OPN-10	ACAACTGGGG	OPN-10	OPN-10	395
OPN-11	TCGCCGCAAA	OPN-11	OPN-11	283
OPN-12	CACAGACACC	OPN-12	OPN-12	206, 241, 676, 307, 322, 681, 802, 278, 899, 530, 444, 522, 927
OPN-13	AGCGTCACTC	OPN-13	OPN-13	233
OPN-14	TCGTGCGGGT	OPN-14		
OPN-15	CAGCGACTGT	OPN-15		
OPN-16	AAGCGACCTG			
OPN-17	CATTGGGGAG			
OPN-18	GGTGAGGTCA			
OPN-19	GTCCGTACTG	OPN-19		
OPN-20	GGTGCTCCGT	OPN-20	OPN-20	238, 237, 413, 236
OPP-01	GTAGCACTCC		OPP-01	
OPP-02	TCGGCACGCA	OPP-02	OPP-02	
OPP-03	CTGATACGCC		OPP-03	230, 235, 520
OPP-04	GTGTCTCAGG		OPP-04	436
OPP-05	CCCCGGTAAC	OPP-05	OPP-05	
OPP-06	GTGGGCTGAC	OPP-06	OPP-06	
OPP-07	GTCCATGCCA			

OPP-08	ACATCGCCCA	OPP-08		
OPP-09	GTGGTCCGCA	OPP-09	OPP-09	517
OPP-10	TCCCGCCTAC	OPP-10	OPP-10	
OPP-11	AACGCGTCGG	OPP-11		
OPP-12	AAGGGCGAGT	OPP-12	OPP-12	
OPP-13	GGAGTGCCTC		OPP-13	254, 636, 379, 378, 301
OPP-14	CCAGCCGAAC		OPP-14	1265
OPP-15	GGAAGCCAAC		OPP-15	417
OPP-16	CCAAGCTGCC		OPP-16	
OPP-17	TGACCCGCCT	OPP-17		
OPP-18	GGCTTGGCCT	OPP-18		
OPP-19	GGGAAGGACA		OPP-19	514, 223, 222
OPP-20	GACCCTAGTC	OPP-20	OPP-20	443
OPQ-01	GGGACGATGG		OPQ-01	1533
OPQ-02	TCTGTCGGTC		OPQ-02	500
OPQ-03	GGTCACCTCA			
OPQ-04	AGTGCGCTGA	OPQ-04		
OPQ-05	CCGCGTCTTG	OPQ-05	OPQ-05	
OPQ-06	GAGCGCCTTG	OPQ-06		
OPQ-07	CCCCGATGGT		OPQ-07	
OPQ-08	CTCCAGCGGA	OPQ-08	OPQ-08	201
OPQ-09	GGCTAACCGA	OPQ-09	OPQ-09	
OPQ-10	TGTGCCCGAA	OPQ-10		
OPQ-11	TCTCCGCAAC	OPQ-11		
OPQ-12	AGTAGGGCAC	OPQ-12		
OPQ-13	GGAGTGGACA	OPQ-13	OPQ-13	291, 292
OPQ-14	GGACGCTTCA	OPQ-14		
OPQ-15	GGGTAACGTG	OPQ-15	OPQ-15	
OPQ-16	AGTGCAGCCA		OPQ-16	357
OPQ-17	GAAGCCCTTG	OPQ-17	OPQ-17	308, 265
OPQ-18	AGGCTGGGTG		OPQ-18	219, 372
OPQ-19	CCCCCTATCA	OPQ-19	OPQ-19	549
OPQ-20	TCGCCCAGTC	OPQ-20	OPQ-20	378
OPR-01	TGCGGGTCCT	OPR-01		
OPR-02	CACAGCTGCC		OPR-02	1188
OPR-03	ACACAGAGGG	OPR-03	OPR-03	772, 240, 805, 401, 420, 300

OPR-04	CCCGTAGCAC	OPR-04	OPR-04	
OPR-05	GACCTAGTGG	OPR-05		
OPR-06	GTCTACGGCA	OPR-06		
OPR-07	ACTGGCCTGA	OPR-07	OPR-07	201
OPR-08	CCCGTTGCCT			
OPR-09	TGAGCACGAG	OPR-09	OPR-09	1292
OPR-10	CCATTCCCCA	OPR-10	OPR-10	533, 255
OPR-11	GTAGCCGTCT	OPR-11		
OPR-12	ACAGGTGCGT	OPR-12		
OPR-13	GGACGACAAG	OPR-13	OPR-13	
OPR-14	CAGGATTCCC	OPR-14	OPR-14	
OPR-15	GGACAACGAG	OPR-15	OPR-15	
OPR-16	CTCTGCGCGT	OPR-16	OPR-16	251, 412, 1257
OPR-17	CCGTACGTAG	OPR-17	OPR-17	396
OPR-18	GGCTTTGCCA			
OPR-19	CCTCCTCATC	OPR-19	OPR-19	391, 240, 412, 775, 206, 241, 589, 406, 450, 560, 711, 477
OPR-20	ACGGCAAGGA		OPR-20	215, 320
OPS-01	CTACTGCGCT	OPS-01	OPS-01	701, 952, 816
OPS-02	CCTCTGACTG	OPS-02	OPS-02	330
OPS-03	CAGAGGTCCC		OPS-03	282
OPS-04	CACCCCCTTG	OPS-04	OPS-04	272, 394
OPS-05	TTTGGGGCCT	OPS-05	OPS-05	
OPS-06	GATACCTCGG	OPS-06		
OPS-07	TCCGATGCTG		OPS-07	604
OPS-08	TTCAGGGTGG			
OPS-09	TCCTGGTCCC		OPS-09	
OPS-10	ACCGTTCCAG	OPS-10		
OPS-11	AGTCGGGTGG			
OPS-12	CTGGGTGAGT			
OPS-13	GTCGTTCCTG	OPS-13		
OPS-14	AAAGGGGTCC	OPS-14	OPS-14	460
OPS-15	CAGTTCACGG	OPS-15		
OPS-16	AGGGGGTTCC		OPS-16	
OPS-17	TGGGGACCAC			
OPS-18	CTGGCGAACT			
OPS-19	GAGTCAGCAG			
OPS-20	TCTGGACGGA		OPS-20	

OPX-01	CTGGGCACGA		OPX-01	200
OPX-02	TTCCGCCACC		OPX-02	
OPX-03	TGGCGCAGTG	OPX-03		
OPX-04	CCGCTACCGA	OPX-04	OPX-04	
OPX-05	CCTTTCCCTC	OPX-05	OPX-05	384, 217, 309, 460, 445, 474, 278, 380, 223
OPX-06	ACGCCAGAGG	OPX-06	OPX-06	
OPX-07	GAGCGAGGCT		OPX-07	772, 363, 297
OPX-08	CAGGGGTGGA		OPX-08	304, 236
OPX-09	GGTCTGGTTG		OPX-09	
OPX-10	CCCTAGACTG	OPX-10		
OPX-11	GGAGCCTCAG		OPX-11	321, 227, 235
OPX-12	TCGCCAGCCA		OPX-12	336
OPX-13	ACGGGAGCAA		OPX-13	
OPX-14	ACAGGTGCTG			
OPX-15	CAGACAAGCC			
OPX-16	CTCTGTTCGG	OPX-16	OPX-16	251, 332
OPX-17	GACACGGACC		OPX-17	
OPX-18	GACTAGGTGG	OPX-18		
OPX-19	TGGCAAGGCA		OPX-19	689, 486
OPX-20	CCCAGCTAGA	OPX-20	OPX-20	
OPY-01	GTGGCATCTC		OPY-01	
OPY-02	CATCGCCGCA	OPY-02	OPY-02	259, 211
OPY-03	ACAGCCTGCT	OPY-03		
OPY-04	GGCTGCAATG	OPY-04	OPY-04	291, 703
OPY-05	GGCTGCGACA	OPY-05	OPY-05	660, 475
OPY-06	AAGGCTCACC	OPY-06		
OPY-07	AGAGCCGTCA	OPY-07	OPY-07	546
OPY-08	AGGCAGAGCA	OPY-08	OPY-08	
OPY-09	AGCAGCGCAC	OPY-09	OPY-09	235
OPY-10	CAAACGTGGG	OPY-10		
OPY-11	AGACGATGGG		OPY-11	612
OPY-12	AAGCCTGCGA		OPY-12	237, 423
OPY-13	GGGTCTCGGT			
OPY-14	GGTCGATCTG		OPY-14	
OPY-15	AGTCGCCCTT	OPY-15	OPY-15	359
OPY-16	GGGCCAATGT	OPY-16		

OPY-17	GACGTGGTGA	OPY-17	OPY-17	
OPY-18	GTGGAGTCAG		OPY-18	235
OPY-19	TGAGGGTCCC		OPY-19	203, 235
OPY-20	AGCCGTGGAA			
OPZ-01	TCTGTGCCAC	OPZ-01	OPZ-01	
OPZ-02	CCTACGGGGA		OPZ-02	
OPZ-03	CAGCACCGCA	OPZ-03	OPZ-03	204
OPZ-04	AGGCTGTGCT	OPZ-04	OPZ-04	373, 200, 286, 253
OPZ-05	TCCCATGCTG	OPZ-05	OPZ-05	406
OPZ-06	GTGCCGTTCA	OPZ-06	OPZ-06	
OPZ-07	CCAGGAGGAC	OPZ-07	OPZ-07	516, 253, 250
OPZ-08	GGGTGGGTAA	OPZ-08		
OPZ-09	CACCCCAGTC		OPZ-09	
OPZ-10	CCGACAAACC	OPZ-10		
OPZ-11	CTCAGTCGCA	OPZ-11		
OPZ-12	TCAACGGGAC	OPZ-12	OPZ-12	
OPZ-13	GACTAAGCCC	OPZ-13		
OPZ-14	TCGGAGGTTC	OPZ-14	OPZ-14	
OPZ-15	CAGGGCTTTC	OPZ-15		
OPZ-16	TCCCCATCAC	OPZ-16	OPZ-16	315, 657, 418, 220, 417
OPZ-17	CCTTCCCACT	OPZ-17	OPZ-17	330, 300
OPZ-18	AGGGTCTGTG	OPZ-18	OPZ-18	
OPZ-19	GTGCGAGCAA	OPZ-19	OPZ-19	321
OPZ-20	ACTTTGGCGG	OPZ-20	OPZ-20	470, 241, 239, 235
**Total number sequences**				
**400**		**258**	**271**	

Table S2.**Sequences of iSCAR Primers for oil palm**

No.	Sequence ID	Primer Name	Product Size	Forward Primer	Reverse Primer
1)	>CN599373	[OPM-01]	304	[GTTGGTGGAGGTGATGAT]	[CCACCAACAGCANNGAAG]
2)	>CN599454	[OPI-16]	351	[TCTCCGCCAGCCGCACCT]	[GGCGGAGATGCCGAAAGA]
3)	>CN599553	[OPN-08]	366	[ACCTCAGCATCAGCTGCT]	[AGCTGAGGTCGATAGGAAT]
4)	>CN600042	[OPJ-18]	235	[TGGTCGCACACTGGCTGA]	[TGCGACCATCCNCAAGTT]
5)	>CN600095	[OPD-17]	269	[TTTCCCACACATAATAAC]	[GTGGGAAAAAGGTCCCCA]
6)	>CN600235	[OPL-03]	235	[CCAGCAGCGTCTGATCTT]	[GCTGCTGGTCCNNNGGAA]
7)	>CN600372	[OPP-19]	222	[GGGAAGGATTACGGCAGA]	[TCCTTCCCCATGAACTAT]
8)	>CN600396	[OPF-14]	401	[TGCTGCAGCCACCGCTCC]	[CCTGCAGCANNGAGCATGG]
9)	>CN600609	[OPD-02]	283	[GGACCCAATGTTCGAGTT]	[TTGGGTCCATCTCTGCAC]
10)	>CN600851	[OPQ-18]	219	[AGGCTGGGGAAGGCAACA]	[CCCAGCCTGAAGTAAGGG]
11)	>CN601059	[OPY-02]	211	[CATCGCCGAGATCGTCTC]	[CGGCGATGCCGGAGGCGG]
12)	>CN601253	[OPB-15]	211	[GGAGGGTGGCCGGCACTC]	[CACCCTCCNNGATGGCAA]
13)	>CN601454	[OPD-14]	258	[CTTCCCCAATAGAAGTTGT]	[TGGGGAAGAAGAGCGGAA]
14)	>CN601772	[OPP-13]	301	[GGAGTGCCGGGAGAGCCG]	[GGCACTCCCGCTTGCNCT]
15)	>DW247851	[OPN-08]	366	[ACCTCAGCATCAGCTGCT]	[AGCTGAGGTCGATAGGAAT]
16)	>DW247968	[OPL-15]	343	[AAGAGAGGATGAGCATGC]	[CCTCTCTTCGAGCTCTGT]
17)	>DW248174	[OPF-14]	201	[TGCTGCAGTTCCAACGTT]	[CTGCAGCAGGGAGGATCA]
18)	>DW248218	[OPN-08]	366	[ACCTCAGCATCAGCTGCT]	[AGCTGAGGTCGATAGGAAT]
19)	>DW248238	[OPN-08]	262	[ACCTCAGCAGCTACAGTC]	[GCTGAGGTAAGGTCTTAA]
20)	>DW248263	[OPG-20]	330	[TCTCCCTCCTCCTCCTCC]	[GAGGGAGAGCTGCAGTAC]
21)	>DW248319	[OPI-02]	296	[GGAGGAGATGTACGCGGA]	[TCTCCTCCAGATATAATG]
22)	>DW248359	[OPF-14]	201	[TGCTGCAGTTCCAACGTT]	[CTGCAGCAGGGAGGATCA]
23)	>DW248473	[OPI-02]	296	[GGAGGAGATGTACGCGGA]	[TCTCCTCCAGATATAATG]
24)	>DW248528	[OPE-18]	436	[GGACTGCATGGTCATTTG]	[TGCAGTCCCACCAGATCC]
25)	>DW248694	[OPG-20]	330	[TCTCCCTCCTCCTCCTCC]	[GAGGGAGAGCTGCAGTAC]
26)	>DW248739	[OPD-04]	430	[TCTGGTGAAGTTCAAGGA]	[TCACCAGAGGACAACACC]
27)	>EB643525	[OPF-03]	224	[CCTGATCAAATCAGGGAC]	[TGATCAGGATTTTATATC]
28)	>EB643541	[OPF-03]	224	[CCTGATCAAATCAGGGAC]	[TGATCAGGATTTTATATC]
29)	>EB643542	[OPF-03]	224	[CCTGATCAAATCAGGGAC]	[TGATCAGGATTTTATATC]
30)	>EB643543	[OPF-03]	224	[CCTGATCAAATCAGGGAC]	[TGATCAGGATTTTATATC]
31)	>EB643546	[OPF-03]	224	[CCTGATCAAATCAGGGAC]	[TGATCAGGATTTTATATC]
32)	>EB643548	[OPF-03]	224	[CCTGATCAAATCAGGGAC]	[TGATCAGGATTTTATATC]
33)	>EB643549	[OPF-03]	224	[CCTGATCAAATCAGGGAC]	[TGATCAGGATTTTATATC]
34)	>EB643552	[OPF-03]	224	[CCTGATCAAATCAGGGAC]	[TGATCAGGATTTTATATC]
35)	>EL563720	[OPQ-13]	291	[GGAGTGGAAGGCGTGAAA]	[TCCACTCCTTTGATTTAC]
36)	>EL563729	[OPD-14]	245	[CTTCCCCAAGACGCCCGAGC]	[TTGGGGAAGTTGACCTTGG]
37)	>EL563735	[OPB-03]	235	[CATCCCCCCAGACCAGCA]	[GGGGGATGCCCTCCTTGT]
38)	>EL563739	[OPQ-13]	292	[GGAGTGGAAGGCGTGAAA]	[TCCACTCCTTTGATTTTA]
39)	>EL595430	[OPI-02]	287	[GGAGGAGAAGAAGGAGAG]	[CTCTCCTCCACCTTCTCCA]
40)	>EL595437	[OPK-02]	235	[GTCTCCGCGGTGGCATGC]	[GCGGAGACGGAGGACGAG]
41)	>EL595443	[OPQ-08]	201	[CTCCAGCGGCTCGTGATGC]	[CGCTGGAGTGGGAGCCAT]
42)	>EL595471	[OPK-02]	235	[GTCTCCGCGGTGGCATGC]	[GCGGAGACGGAGGACGAG]
43)	>EL595518	[OPB-03]	235	[CATCCCCCTGACCAGCA]	[GGGGGATGCCCTCCTTGT]
44)	>EL595551	[OPI-14]	206	[TGACGGCGGATTCCTCGAG]	[CCGCCGTCACCTTCGGCCG]
45)	>EL595569	[OPD-14]	245	[CTTCCCCAAGACGCCCGAGC]	[TTGGGGAAGTTGACCTTGG]
46)	>EL608661	[OPZ-04]	200	[AGGCTGTGCCGGATTCCCT]	[CACAGCCTTCACCGTGCT]
47)	>EL608721	[OPG-20]	497	[TCTCCCTCGCCCTCGCCA]	[TGAGGGAGACGAAGGTGTT]
48)	>EL608778	[OPM-01]	304	[GTTGGTGGTGGTGATGAT]	[CCACCAACAGCATTGAAG]
49)	>EL608794	[OPP-03]	235	[CTGATACGATTGATAATG]	[CGTATCAGAGCTCTCAAC]
50)	>EL608801	[OPG-20]	333	[TCTCCCTCCTCCTCCTCC]	[GAGGGAGAGCTGCAGTAC]
51)	>EL608823	[OPQ-13]	292	[GGAGTGGAAGGCGTGAAA]	[TCCACTCCTTTGATTTTA]

52) >EL608839 [OPQ-13] 292 [GGAGTGGAAGGCGTGAAA] [TCCACTCCTTTGATTTTA]
53) >EL608885 [OPI-02] 211 [GGAGGAGAAGAAGGAAGA] [TCTCCTCCTCTTGATCAC]
54) >EL608906 [OPB-03] 235 [CATCCCCCCAGACCAGCA] [GGGGGATGCCCTCCTTGT]
55) >EL608922 [OPY-05] 475 [GGCTGCGATGGCTACGGG] [GTCGCAGCCAGCAACGCGC]
56) >EL608940 [OPF-05] 393 [CCGAATTCGGAGGATTGA] [GGAATTCGGTTCAATCCCT]
57) >EL681015 [OPR-16] 251 [CTCTGCGCGCCCACGGGAG] [ACGCGCAGAGACTATGAGTA]
58) >EL681019 [OPI-03] 315 [CAGAAGCCCCGGCCGCCCC] [GGCTTCTGGGCTTCAGGA]
59) >EL681039 [OPI-02] 476 [GGAGGAGACGGAGGCGGC] [CTCTCCTCCTCTTCTCAGC]
60) >EL681048 [OPB-03] 417 [CATCCCCCCTTCCACTCC] [GGGGGATGTCGTCGAGGC]
61) >EL681135 [OPL-11] 704 [ACGATGAGTGGTGATTCG] [CTCATCGTGTGATCACNC]
62) >EL681138 [OPB-06] 406 [TGCTCTGCTATAATCGTA] [GCAGAGCATGCTAGTATG]
63) >EL681142 [OPS-01] 701 [CTACTGCGCGCTTGCNCGC] [CGCAGTAGGTTGNCGGTC]
64) >EL681143 [OPJ-11] 1100 [ACTCCTGCGATAGCTCACTG] [GCAGGAGTGGTGTCATGA]
65) >EL681145 [OPR-02] 1188 [CACAGCTGANGTGCATGA] [CAGCTGTGNTACACACAA]
66) >EL681146 [OPR-17] 396 [CCGTACGTCACACGTCAA] [ACGTACGGTGTCTCGCAC]
67) >EL681147 [OPA-14] 266 [TCTGTGCTGTACGCGCGTA] [AGCACAGAGCGTNTCNGT]
68) >EL681150 [OPL-04] 943 [GACTGCACGTGATCGCGC] [TGTGCAGTCTGATATGCAC]
69) >EL681151 [OPP-03] 520 [CTGATACGGCGTGATCTA] [CGTATCAGTCTCTCGCGT]
70) >EL681155 [OPL-11] 1908 [ACGATGAGATGATGACGT] [CTCATCGTGCGTATCCGC]
71) >EL681160 [OPL-11] 938 [ACGATGAGAACAGGATTG] [CTCATCGTCACATCGTCT]
72) >EL681162 [OPR-16] 412 [CTCTGCGCACTGACTACT] [ACGCGCAGAGCGAGCATCAT]
73) >EL681163 [OPE-17] 1067 [CTACTGCCAGAATACATC] [GGCAGTAGATCAGTACGG]
74) >EL681164 [OPL-04] 616 [GACTGCACGACAGACACG] [GTGCAGTCNTCTCACGTC]
75) >EL681166 [OPS-01] 816 [CTACTGCGCAGTGCGCATC] [CGCAGTAGTGCATGATGA]
76) >EL681170 [OPG-04] 353 [AGCGTGTCGCTGCATGGC] [GACACGCTCGTCGTCTCT]
77) >EL681171 [OPR-09] 1292 [TGAGCACGTTATCAGCTC] [TCGTGCTCATGTGTTGTGA]
78) >EL681172 [OPA-08] 1457 [GTGACGTAGCTGCTAGCGA] [TACGTCACGTCTNTTATG]
79) >EL681176 [OPB-06] 282 [TGCTCTGCATGCATATGT] [GCAGAGCACAAGTATCGT]
80) >EL681178 [OPI-10] 648 [ACAACGCGAATATGTCGAT] [CGCGTTGTCNAGCATANT]
81) >EL681180 [OPA-10] 697 [GTGATCGCTACACTATGC] [GCGATCACGTTACGATGT]
82) >EL681183 [OPF-11] 1307 [TTGGTACCATGCCGCAGT] [GGTACCAACTGTGTGCGT]
83) >EL681185 [OPE-04] 1128 [GTGACATGATGTGCAAAT] [CATGTCACGTGATAGGNC]
84) >EL681217 [OPI-16] 406 [TCTCCGCCGACAGGTTCA] [GGCGGAGATGGCGAGAGT]
85) >EL681245 [OPE-13] 663 [CCCGATTCTAGGGCCGTT] [GAATCGGGACCTCCATCA]
86) >EL681288 [OPB-04] 326 [GGACTGGATTCTCCCTCC] [TCCAGTCCAGACCGGGCC]
87) >EL681331 [OPI-16] 574 [TCTCCGCCTCACTCGGAC] [GGCGGAGATATCAAGGTC]
88) >EL681352 [OPI-09] 283 [TGGAGAGCTCTTCGCCCG] [GCTCTCCATAGTTCGGCG]
89) >EL681403 [OPF-19] 612 [CCTCTAGAACCCAGAAAG] [TCTAGAGGCAAACAACCT]
90) >EL681409 [OPK-17] 369 [CCCAGCTGGACCATCAAC] [CACAGCTGGGGTCTTCGTCC]
91) >EL681430 [OPR-19] 240 [CCTCCTCATCCATGAGGGCG] [GATGAGGAGGGCCCCGCTGG]
92) >EL681475 [OPZ-16] 657 [TCCCCATCTGTCCTACTG] [GATGGGGAGTTCTCGAAT]
93) >EL681632 [OPA-13] 423 [CAGCACCCTATGCCTCTG] [GGGTGCTGGCCTGCTGCT]
94) >EL681643 [OPJ-05] 762 [CTCCATGGTTCGTCCTTG] [CCATGGAGGTGTAGTAGC]
95) >EL681702 [OPG-13] 515 [CTCTCCGCGTCCTCTAAT] [GCGGAGAGCGGCCATGAG]
96) >EL681749 [OPI-02] 587 [GGAGGAGAGGGTCTCCATGG] [TCTCCTCCAACAGGAAAG]
97) >EL681751 [OPI-16] 298 [TCTCCGCCCTCCGCCTCCCC] [GGCGGAGAAAGCGCCGAA]
98) >EL681799 [OPM-01] 304 [GTTGGTGGTGGTGATGAT] [CCACCAACAGCATTGAAG]
99) >EL681813 [OPZ-16] 315 [TCCCCATCAAACTAGTTCT] [GATGGGGAAGCAGAGTGA]
100) >EL681828 [OPF-06] 451 [GGGAATTCGAGTTCTAGCG] [GAATTCCCAAATTCTCCA]
101) >EL681849 [OPM-02] 244 [ACAACGCCAACCGGGACA] [GGCGTTGTGGATGCTCTC]
102) >EL681873 [OPM-20] 245 [AGGTCTTGTCTGCCTTGA] [CAAGACCTGAACCAAAAA]
103) >EL681899 [OPB-10] 486 [CTGCTGGGTAGTTACTTC] [GTCCCAGCAGACGGCCAACA]
104) >EL681916 [OPX-08] 236 [CAGGGGTGGCATGCAGATC] [CCACCCCTGAGCCGCAGAA]
105) >EL681934 [OPG-20] 330 [TCTCCCTCCTCCTCCTCC] [GAGGGAGAGCTGCAGTAC]
106) >EL681952 [OPI-03] 266 [CAGAAGCCCCGGCCGCCCC] [GGCTTCTGGGCTTCAGGA]
107) >EL681991 [OPG-20] 330 [TCTCCCTCCTCCTCCTCC] [GAGGGAGAGCTGCAGTAC]

108)	>EL682053	[OPI-02]	241	[GGAGGAGAGAAAAGAACGG]	[TCTCCTCCTCCTTCACGT]
109)	>EL682156	[OPI-16]	643	[TCTCCGCCGACGACCTTC]	[GGCGGAGAAGGTTGGGCT]
110)	>EL682189	[OPF-05]	530	[CCGAATTCTGCCGCCCCG]	[GAATTCGGCACGAGGAGA]
111)	>EL682200	[OPI-02]	367	[GGAGGAGAAGCTGAGGCG]	[TCTCCTCCTGTGCAATAT]
112)	>EL682214	[OPF-16]	316	[GGAGTACTTGGAAGAAGA]	[AGTACTCCTCATCAGTAA]
113)	>EL682225	[OPI-16]	211	[TCTCCGCCGCCACCGCCT]	[GGCGGAGAGGGCGATGAG]
114)	>EL682227	[OPR-20]	320	[ACGGCAAGCCCATCACTC]	[CCTTGCCGTGCGCCATGTC]
115)	>EL682250	[OPG-13]	515	[CTCTCCGCGTCCTCTAAT]	[GCGGAGAGCGGCCATGAG]
116)	>EL682275	[OPK-17]	369	[CCCAGCTGGACCATCAAC]	[CACAGCTGGGGTCTTCGTCC]
117)	>EL682292	[OPD-09]	299	[CTCTGGAGCATGATTACA]	[CTCCAGAGAACCCCCACT]
118)	>EL682337	[OPP-15]	417	[GGAAGCCATGCTTTTCCT]	[TGGCTTCCAGTGGCGCTT]
119)	>EL682398	[OPP-09]	517	[GTGGTCCGTCATCATCAC]	[CGGACCACGACCGTAGCG]
120)	>EL682422	[OPF-14]	634	[TGCTGCAGCACGTGCTAT]	[CTGCAGCATGAGAGTTCT]
121)	>EL682445	[OPM-01]	466	[GTTGGTGGGCACACCAAA]	[CCACCAACAATAAACCAG]
122)	>EL682534	[OPM-13]	289	[GGTGGTCAGAATCCAAAG]	[TGACCACCAGTCATGCCG]
123)	>EL682551	[OPK-16]	519	[GAGCGTCGTCAGGACCAG]	[CGACGCTCTGTAAATGGA]
124)	>EL682566	[OPD-09]	471	[CTCTGGAGACATCTAGAACA]	[CTCCAGAGTAGGGTCGTG]
125)	>EL682609	[OPX-07]	772	[GAGCGAGGTGTTCCTGCA]	[CCTCGCTCGTAGATAATG]
126)	>EL682714	[OPP-15]	417	[GGAAGCCATGCTTTTCCT]	[TGGCTTCCAGTGGCGCTT]
127)	>EL682718	[OPX-11]	235	[GGAGCCTCANAAGTTCTCA]	[GAGGCTCCTTGTGTGACT]
128)	>EL682897	[OPB-15]	420	[GGAGGGTGGTGCCAGACG]	[CACCCTCCAGTGTCAGCC]
129)	>EL682982	[OPP-03]	230	[CTGATACGATTGATTAAT]	[CGTATCAGAGTTTCAACT]
130)	>EL683063	[OPG-02]	223	[GGCACTGAAGGCAATTTT]	[TCAGTGCCTCCATGCCTG]
131)	>EL683247	[OPF-14]	201	[TGCTGCAGTTCCAACGTT]	[CTGCAGCAGGGAGGATCA]
132)	>EL683299	[OPM-03]	277	[GGGGGATGCNGACGATGA]	[CATCCCCCTTNTCAGTTG]
133)	>EL683594	[OPX-01]	200	[CTGGGCACACCGGCAGTG]	[GTGCCCAGACCTGTGGTC]
134)	>EL683985	[OPG-06]	369	[GTGCCTAATATCCGATTA]	[GTTAGGCACTTGAGTAACA]
135)	>EL684054	[OPI-02]	212	[GGAGGAGAACGTGTACAT]	[CTCTCCTCCTTCTGCTCGA]
136)	>EL684084	[OPI-06]	504	[AAGGCGGCCATCGATTCC]	[CTGCCGCCTTTGCTGCAGCT]
137)	>EL684085	[OPI-02]	408	[GGAGGAGAGTTGGCTTACC]	[TCTCCTCCAGATGATCAT]
138)	>EL684090	[OPI-02]	408	[GGAGGAGAGTTGGCTTACC]	[TCTCCTCCAGATGATCAT]
139)	>EL684124	[OPB-06]	211	[TGCTCTGCTTACTACAAC]	[GCAGAGCAACATCCAATT]
140)	>EL684225	[OPY-05]	660	[GGCTGCGACGCATCAGTAA]	[TCGCAGCCCAGAAGTTGA]
141)	>EL684269	[OPB-10]	200	[CTGCTGGGGAGTACATGA]	[TCCCAGCAGCCAATGCAGT]
142)	>EL684310	[OPM-01]	304	[GTTGGTGGTGGTGATGAT]	[CCACCAACAGCATTGAAG]
143)	>EL684340	[OPM-01]	532	[GTTGGTGGTGGCTGCAGG]	[CCACCAACCTGCCATTGC]
144)	>EL684346	[OPG-20]	508	[TCTCCCTCGCTCCCCACC]	[GAGGGAGAGGGACTCGGA]
145)	>EL684360	[OPD-14]	468	[CTTCCCCAATTTCCCTACC]	[CTTGGGGAAGGAGAGGTCCT]
146)	>EL684442	[OPM-01]	532	[GTTGGTGGTGGCTGCAGG]	[CCACCAACCTGCCATTGC]
147)	>EL684511	[OPM-01]	304	[GTTGGTGGTGGTGATGAT]	[CCACCAACAGCATTGAAG]
148)	>EL684532	[OPN-08]	330	[ACCTCAGCATCAGCTGCT]	[AGCTGAGGTCGATAGGAAC]
149)	>EL684560	[OPJ-05]	295	[CTCCATGGATGAGGATCA]	[CCATGGAGTCAACCCCAC]
150)	>EL684589	[OPY-05]	660	[GGCTGCGACGCATCAGTAA]	[TCGCAGCCCAGAAGTTGA]
151)	>EL684595	[OPI-02]	381	[GGAGGAGAAAAGACTCAA]	[CCTCTCCTCCTCCTCATCGT]
152)	>EL684612	[OPP-19]	222	[GGGAAGGATTACGGCAGA]	[TCCTTCCCCATGAACTAT]
153)	>EL684736	[OPE-07]	386	[AGATGCAGAAGAACTCGT]	[CTGCATCTCAGCTGTACT]
154)	>EL684789	[OPG-18]	410	[GGCTCATGTAAGGGGTCAT]	[ACATGAGCCCAAGTCTTCC]
155)	>EL684817	[OPC-01]	537	[TTCGAGCCAAAGGCGGCGC]	[GGCTCGAACACGCCAATC]
156)	>EL684915	[OPI-02]	208	[GGAGGAGAATGCTGCCTC]	[TCTCCTCCATTGCTCTCG]
157)	>EL684954	[OPB-13]	235	[TTCCCCCGGACCAGCAGA]	[CGGGGGAATTCCCTCCTT]
158)	>EL684957	[OPP-19]	222	[GGGAAGGACTATGGCAGGC]	[TCCTTCCCCATGAGCTGT]
159)	>EL685108	[OPG-11]	307	[TGCCCGTCCTGCCCGAAC]	[GACGGGCAGGTCGAGTGT]
160)	>EL685135	[OPR-10]	255	[CCATTCCCTACCGCGACC]	[GGGGAATGGTATCAGGCAT]
161)	>EL685145	[OPC-17]	433	[TTCCCCCCAACCTGGGGCC]	[GGGGGGAAAAAAAAAGGT]
162)	>EL685158	[OPC-17]	399	[TTCCCCCCCCAAAAATAT]	[GGGGGGAAAAACCCCCCA]
163)	>EL685161	[OPP-04]	436	[GTGTCTCATCAACATTCA]	[TGAGACACACCTGCCTTC]
164)	>EL685166	[OPD-09]	299	[CTCTGGAGCATGATTACA]	[CTCCAGAGAACCCCCACT]

165) >EL685175	[OPM-01]304	[GTTGGTGGTGGTGATGAT]	[CCACCAACAGCATTGAAG]
166) >EL685216	[OPP-19] 222	[GGGAAGGACTATGGCAGGC]	[TCCTTCCCCATGAGCTGT]
167) >EL685224	[OPG-20] 327	[TCTCCCTCCTGCTCCTGC]	[GAGGGAGAGCTGCAGTAC]
168) >EL685435	[OPM-01]304	[GTTGGTGGTGGTGATGAT]	[CCACCAACAGCATTGAAG]
169) >EL685550	[OPM-01]304	[GTTGGTGGTGGTGATGAT]	[CCACCAACAGCATTGAAG]
170) >EL685558	[OPX-05] 384	[CCTTTCCCCAATAGAGGA]	[AGGGAAAGGCCTCTTATAA]
171) >EL685571	[OPA-08]319	[GTGACGTACGTTGTTCAC]	[TACGTCACGACGTGTACA]
172) >EL685624	[OPM-01]304	[GTTGGTGGTGGTGATGAT]	[CCACCAACAGCATTGAAG]
173) >EL685672	[OPM-01]304	[GTTGGTGGTGGTGATGAT]	[CCACCAACAGCATTGAAG]
174) >EL685864	[OPR-20] 320	[ACGGCAAGCCCATCACTC]	[CCTTGCCGTGCGCCATGTC]
175) >EL685909	[OPR-03] 401	[ACACAGAGGCCTCAAGAGA]	[CTCTGTGTTTTCTTTTCC]
176) >EL686132	[OPC-16] 715	[CACACTCCACACAAGAGTT]	[GGAGTGTGATTTTATTTT]
177) >EL686140	[OPL-15] 342	[AAGAGAGGACTCGAAGCG]	[CCTCTCTTCTCCTGCACC]
178) >EL686213	[OPM-01]304	[GTTGGTGGTGGTGATGAT]	[CCACCAACAGCATTGAAG]
179) >EL686218	[OPM-01]304	[GTTGGTGGTGGTGATGAT]	[CCACCAACAGCAGTGAAG]
180) >EL686324	[OPI-12] 459	[AGAGGGCACTGCGAATTCC]	[TGCCCTCTCAATCCACAA]
181) >EL686349	[OPP-19] 222	[GGGAAGGATTACGGCAGA]	[TCCTTCCCCATGAACTAT]
182) >EL686394	[OPX-16] 251	[CTCTGTTCTGGCACCGGT]	[GAACAGAGCAATAGAAAC]
183) >EL686412	[OPD-15] 408	[CATCCGTGGGGGGAGCAA]	[CACGGATGCAGTCTAGCA]
184) >EL686440	[OPY-15] 359	[AGTCGCCCCCGATGTCTC]	[GGGCGACTTCCGCCTTCC]
185) >EL686479	[OPF-10] 510	[GGAAGCTTTTGCCTGAGG]	[AAGCTTCCAGAAGCAGGT]
186) >EL686507	[OPJ-18] 235	[TGGTCGCACACTGGCTGA]	[TGCGACCATCCTCAAGTT]
187) >EL686508	[OPP-13] 636	[GGAGTGCCTCGATTGTCATT]	[AGGCACTCCTCCTGTGTTG]
188) >EL686524	[OPM-01]304	[GTTGGTGGTGGTGATGAT]	[CCACCAACAGCATTGAAG]
189) >EL686545	[OPC-10] 402	[TGTCTGGGATATGTTAAA]	[CCCAGACACCACAAGCTG]
190) >EL686616	[OPM-11] 419	[GTCCACTGGTGTCTTCAC]	[CAGTGGACACCAAATCCT]
191) >EL686663	[OPE-14] 587	[TGCGGCTGTTCGGCGGTT]	[CAGCCGCAGGCCCATAAG]
192) >EL686685	[OPX-07] 363	[GAGCGAGGACCCGCCCTC]	[CCTCGCTCTGCACCCGGT]
193) >EL686686	[OPY-04] 703	[GGCTGCAAATGCGGCACT]	[TTGCAGCCGCCGTTCTCG]
194) >EL686722	[OPB-03] 235	[CATCCCCCCAGACCAGCA]	[GGGGGATGCCCTCCTTGT]
195) >EL686777	[OPJ-18] 235	[TGGTCGCACACTGGCTGA]	[TGCGACCATCCTCAAGTT]
196) >EL686821	[OPA-13] 435	[CAGCACCCTATGCCACTG]	[GGGTGCTGGCCTGCTGCT]
197) >EL687010	[OPL-15] 266	[AAGAGAGGATCAACTGCT]	[CCTCTCTTCACGGTGCCG]
198) >EL687046	[OPP-19] 223	[GGGAAGGATTACGGCAGA]	[TCCTTCCCCATGAACTAT]
199) >EL687069	[OPA-13] 435	[CAGCACCCTATGCCACTG]	[GGGTGCTGGCCTGCTGCT]
200)>EL687121	[OPI-02] 280	[GGAGGAGATCGACGCTGC]	[TCTCCTCCAAAACAAAAT]
201) >EL687127	[OPG-14] 530	[GGATGAGAAAGCCTGAAG]	[TCTCATCCCCACTCTTGG]
202)>EL687191	[OPD-03] 267	[GTCGCCGTAAATTTCCAC]	[ACGGCGACAACTGGCGGG]
203)>EL687243	[OPF-14] 321	[TGCTGCAGTTTCTACGGA]	[CTGCAGCATCTCGAGCGT]
204)>EL687244	[OPB-08]600	[GTCCACACAGCTGTCTCA]	[GTGTGGACATAGAGAGGC]
205)>EL687367	[OPI-02] 238	[GGAGGAGAGTGATTCATCT]	[TCTCCTCCTTTGTCAACT]
206)>EL687422	[OPJ-18] 235	[TGGTCGCACACTGGCTGA]	[TGCGACCATCCTCAAGTT]
207)>EL687458	[OPZ-03] 204	[CAGCACCGCCTCTTGGCGC]	[GCGGTGCTGCCGTGCCCGC]
208)>EL687489	[OPF-14] 212	[TGCTGCAGCTTTGTAGTG]	[CTGCAGCACCAGATCCAA]
209)>EL687558	[OPE-15] 291	[ACGCACAAGACTTATACT]	[TTGTGCGTGAGCTTTCCT]
210) >EL687564	[OPD-09]299	[CTCTGGAGCATGATTACA]	[CTCCAGAGAACCCCCACT]
211) >EL687598	[OPI-02] 287	[GGAGGAGAAGAAGGAGAG]	[CTCTCCTCCACCTTCTCCA]
212) >EL687697	[OPD-04]235	[TCTGGTGACCCTTCAGCC]	[TCACCAGAAGGAACCTCA]
213) >EL687716	[OPI-02] 381	[GGAGGAGAAAAGACTCAA]	[CCTCTCCTCCTCCTCCTCGT]
214) >EL687838	[OPQ-18] 219	[AGGCTGGGGAAGGCAACA]	[CCCAGCCTGAAGTAAGGG]
215) >EL687862	[OPM-01]304	[GTTGGTGGTGGTGATGAT]	[CCACCAACAGCATTGAAG]
216) >EL688028	[OPY-04] 291	[GGCTGCAAAGCCCTCCAT]	[TTGCAGCCAGAGCCACAA]
217) >EL688094	[OPI-03] 315	[CAGAAGCCCCGGCCGCCCC]	[GGCTTCTGGGCTTCAGGA]
218) >EL688176	[OPQ-20]378	[TCGCCCAGCTCACCCGCG]	[CTGGGCGAGGGGGAGGGA]
219) >EL688253	[OPF-03] 463	[CCTGATCAGCAGCGCCTG]	[TGATCAGGGGGTATGCCC]
220) >EL688264	[OPL-15] 567	[AAGAGAGGACAACGGAAG]	[CCTCTCTTAAAGCTTGCT]
221) >EL688271	[OPL-03] 235	[CCAGCAGCGTCTGATCTT]	[GCTGCTGGTCCGGCGGAA]

222) >EL688273	[OPK-15] 313	[CTCCTGCCGCGAGGGTAT]	[GGCAGGAGGTGCTGCAGC]
223) >EL688344	[OPB-16] 235	[TTTGCCCGAAACGATGGT]	[CGGGCAAAAATTGTAACA]
224) >EL688403	[OPZ-07] 250	[CCAGGAGGTCTCAGGAGC]	[CCTCCTGGCAACTCCAAA]
225) >EL688425	[OPL-03] 235	[CCAGCAGCGTCTGATCTT]	[GCTGCTGGTCCGGCGGAA]
226) >EL688441	[OPZ-04] 373	[AGGCTGTGATTGCTGCCG]	[CACAGCCTTCTCATAGAT]
227) >EL688469	[OPL-03] 235	[CCAGCAGCGTCTGATCTT]	[GCTGCTGGTCCGGCGGAA]
228) >EL688487	[OPF-13] 305	[GGCTGCAGAATCAGCTAAGA]	[CTGCAGCCATCACTTGCT]
229) >EL688513	[OPL-03] 235	[CCAGCAGCGTCTGATCTT]	[GCTGCTGGTCCGGCGGAA]
230) >EL688515	[OPM-01]304	[GTTGGTGGTGGTGATGAT]	[CCACCAACAGCATTGAAG]
231) >EL688528	[OPL-15] 500	[AAGAGAGGATAGCTTAAC]	[CCTCTCTTATCAGATTGC]
232) >EL688535	[OPN-20] 237	[GGTGCTCCGCCTCCGTGGA]	[ACGGAGCACCAGATGGAGGG]
233) >EL688559	[OPL-03] 235	[CCAGCAGCGTCTGATCTT]	[GCTGCTGGTCCGGCGGAA]
234) >EL688573	[OPI-02] 220	[GGAGGAGAAGAAAGGTTT]	[TCTCCTCCCCTCCATTCC]
235) >EL688598	[OPE-18] 440	[GGACTGCATGTGTCGTTT]	[TGCAGTCCCAGCAGATCC]
236) >EL688737	[OPS-07] 604	[TCCGATGCTGTCCCTGCGGA]	[GCATCGGAGAAGAGAGCC]
237) >EL688823	[OPN-20] 238	[GGTGCTCCGCCTCCGTGGA]	[ACGGAGCACCAGATGGAGGG]
238) >EL688899	[OPL-09] 247	[TGCGAGAGGGAGAATCCC]	[CTCTCGCAGCAGCTCCAA]
239) >EL689055	[OPI-02] 297	[GGAGGAGATTCCGTAAAA]	[CTCTCCTCCTGATCTTTCT]
240) >EL689143	[OPL-03] 235	[CCAGCAGCGTCTGATCTT]	[GCTGCTGGTCCGGCGGAA]
241) >EL689146	[OPF-14] 212	[TGCTGCAGCTTTGTAGTG]	[CTGCAGCACCAGATCCAA]
242) >EL689194	[OPI-02] 584	[GGAGGAGAAGCTCATCAT]	[CTCTCCTCCCCCAGAAGGC]
243) >EL689196	[OPB-03] 463	[CATCCCCCCGGACCAGCA]	[GGGGGATGCCCTCTTTAT]
244) >EL689212	[OPQ-18] 372	[AGGCTGGGCATGCAAAGG]	[CCCAGCCTAGTAGCAATC]
245) >EL689303	[OPK-17] 491	[CCCAGCTGGAGCAGTTGA]	[CAGCTGGGACTTCACAGA]
246) >EL689311	[OPE-18] 385	[GGACTGCATCTTCGCTCC]	[TGCAGTCCAGCGAACTCT]
247) >EL689337	[OPF-13] 430	[GGCTGCAGGTGCTTTGTG]	[CTGCAGCCATGGAGAAGA]
248) >EL689352	[OPS-04] 394	[CACCCCCTGAGCAGATAC]	[AGGGGGTGGCTGAGTCTG]
249) >EL689380	[OPX-19] 486	[TGGCAAGGAGTTGGCTTC]	[CCTTGCCAATGTCTAGCA]
250) >EL689383	[OPL-03] 609	[CCAGCAGCCGTCCCAGAT]	[AGCTGCTGGACCAACCCGC]
251) >EL689396	[OPC-19] 403	[GTTGCCAGACTCATTGGT]	[CTGGCAACCTTCCTAAGT]
252) >EL689413	[OPM-18]268	[CACCATCCTCTCCTCCCA]	[GGATGGTGAAATGCGCCA]
253) >EL689508	[OPE-18] 436	[GGACTGCATGGTCATTTG]	[TGCAGTCCCACCAGATCC]
254) >EL689536	[OPK-20] 558	[GTGTCGCGAGCCCTTCCTCC]	[CGCGACACTTGCACACTC]
255) >EL689564	[OPY-07] 546	[AGAGCCGTCGATCGATTGA]	[ACGGCTCTCGAGCGATCT]
256) >EL689573	[OPL-04] 1390	[GACTGCACGTCCATGCGT]	[GTGCAGTCGTATGATGTA]
257) >EL689591	[OPE-07] 781	[AGATGCAGTTACTCAGAT]	[CTGCATCTGTCTAGATGA]
258) >EL689603	[OPN-12] 278	[CACAGACATAGTCACACG]	[TGTCTGTGTATCGTGAGA]
259) >EL689622	[OPN-12] 802	[CACAGACAGACATACACG]	[GTGTCTGTGTAGACTCTGG]
260) >EL689648	[OPJ-08] 1133	[CATACCGTCAGAGACATA]	[ACGGTATGGATCTCAGTC]
261) >EL689650	[OPL-03] 235	[CCAGCAGCGTCTGATCTT]	[GCTGCTGGTCCGGCGGAA]
262) >EL689663	[OPI-07] 394	[CAGCGACATATCACGGAA]	[TGTCGCTGTTCTTACCCA]
263) >EL689664	[OPB-06] 1195	[TGCTCTGCTATCTCCTTT]	[GCAGAGCACGGGCAAGGA]
264) >EL689669	[OPR-03] 240	[ACACAGAGAGAGCGCAAT]	[CTCTGTGTCAGTCTCTGC]
265) >EL689670	[OPG-20] 675	[TCTCCCTCTACTTATCTA]	[GAGGGAGAGAAGGTAGAA]
266) >EL689677	[OPR-03] 300	[ACACAGAGATACTGACAG]	[CTCTGTGTATGTCCATCT]
267) >EL689678	[OPS-01] 952	[CTACTGCGTCAGACATCT]	[GCGCAGTAGACTGTCTCTG]
268) >EL689679	[OPG-20] 284	[TCTCCCTCCTTTTCCCGT]	[GAGGGAGACGGGAGAGAG]
269) >EL689683	[OPX-05] 474	[CCTTTCCCCACCCAGTTT]	[GAGGGAAAGGGAGATAGCCA]
270) >EL689684	[OPN-12] 927	[CACAGACACAGCATACGAG]	[GTGTCTGTGAGTATAGCGA]
271) >EL689688	[OPN-12] 681	[CACAGACATAGAGACTTA]	[TGTCTGTGTGTCGCACAA]
272) >EL689694	[OPA-10] 703	[GTGATCGCTTGCGAAATC]	[GCGATCACTCTGTCAGCG]
273) >EL689705	[OPR-03] 772	[ACACAGAGACATACACAC]	[CTCTGTGTAAGTGTCTGT]
274) >EL689706	[OPR-03] 420	[ACACAGAGATATATCTCT]	[CTCTGTGTATATGTATCT]
275) >EL689709	[OPL-09] 598	[TGCGAGAGAGAGATATAG]	[CTCTCGCATCATATAGAG]
276) >EL689730	[OPK-19] 251	[CACAGGCGCAGCCTCTCT]	[CGCCTGTGAGGGAGCGGA]
277) >EL689756	[OPI-02] 220	[GGAGGAGAAGAAAGGTTT]	[TCTCCTCCCCTCCATTCC]
278) >EL689781	[OPN-12] 530	[CACAGACATCGACTGACA]	[TGTCTGTGTGCGGCACGT]

279) >EL689816	[OPJ-13]	1400	[CCACACTATCTATTACAT]	[TAGTGTGGTAGAGTACAG]
280)>EL689832	[OPC-16]	1617	[CACACTCCCNTTCGCTAG]	[TGGAGTGTGCTCTATGTGG]
281) >EL689852	[OPN-12]	676	[CACAGACAACTCTCACAG]	[TGTCTGTGTATGAATATA]
282) >EL689853	[OPD-17]	546	[TTTCCCACTTCCGGGTGT]	[GTGGGAAAGCCTACAAAG]
283) >EL689877	[OPC-11]	521	[AAAGCTGCTTGGTTGTCT]	[GCAGCTTTAAGTACATGT]
284) >EL689889	[OPL-03]	235	[CCAGCAGCGTCTGATCTT]	[GCTGCTGGTCCGGCGGAA]
285) >EL689907	[OPG-20]	409	[TCTCCCTCAAAACGTCCCA]	[GAGGGAGAGATATGNGCG]
286)>EL690007	[OPR-20]	320	[ACGGCAAGCCCATCACTC]	[CCTTGCCGTGCGCCATGTC]
287) >EL690065	[OPX-16]	251	[CTCTGTTCTGGCACCGGT]	[GAACAGAGCAATAGAAAC]
288)>EL690113	[OPL-19]	223	[GAGTGGTGTAGGAGTATT]	[GTCACCACTCGCTTAGTCCT]
289)>EL690152	[OPM-09]	492	[GTCTTGCGCACTCATTGA]	[CGCAAGACTGCGAGACAC]
290)>EL690157	[OPL-19]	504	[GAGTGGTGGAGTGCGAAC]	[CACCACTCCACGGCAGCT]
291) >EL690163	[OPB-03]	493	[CATCCCCCAGACACCCTC]	[GGGGGATGGTGGTGTGGG]
292) >EL690167	[OPI-07]	856	[CAGCGACACCGAGACGCA]	[TGTCGCTGCATTTGCGCG]
293) >EL690176	[OPB-06]	253	[TGCTCTGCGCTGTCGCTG]	[GCAGAGCACGGACAAACC]
294) >EL690284	[OPL-06]	594	[GAGGGAAGCGAGAGCGAG]	[CTTCCCTCCTCTTCATCT]
295) >EL690318	[OPB-15]	493	[GGAGGGTGGGTGATGTCA]	[ACACCCTCCAGATCCTAGC]
296) >EL690364	[OPC-17]	263	[TTCCCCCCCCCCGGGGGG]	[GGGGGGAAAAAAAAAAAA]
297) >EL690366	[OPX-07]	297	[GAGCGAGGAGGAGGCGGG]	[CCTCGCTCTTCGGTTCTG]
298)>EL690373	[OPP-19]	222	[GGGAAGGACTACGGCAGGC]	[TCCTTCCCCACGAGCTGT]
299)>EL690393	[OPJ-18]	235	[TGGTCGCACACTGGCTGA]	[TGCGACCATCCTCAAGTT]
300)>EL690413	[OPN-08]	330	[ACCTCAGCATCAGCTGCT]	[AGCTGAGGTCGATAGGAAC]
301) >EL690419	[OPG-20]	327	[TCTCCCTCCTGCTCCTGC]	[GAGGGAGAGCTGCAGTAC]
302) >EL690465	[OPN-08]	330	[ACCTCAGCATCAGCTGCT]	[AGCTGAGGTCGATAGGAAC]
303) >EL690505	[OPI-02]	220	[GGAGGAGAAGAAAGGTTT]	[TCTCCTCCCCTCCATTCC]
304)>EL690521	[OPP-19]	222	[GGGAAGGACTACGGCAGGC]	[TCCTTCCCCACGAGCTGT]
305)>EL690528	[OPP-19]	222	[GGGAAGGACTATGGCAGGC]	[TCCTTCCCCATGAGCTGT]
306)>EL690545	[OPP-20]	443	[GACCCTAGCAGATGTTTG]	[CTAGGGTCGATATACTTT]
307) >EL690564	[OPI-02]	212	[GGAGGAGAACGTGTACAT]	[CTCTCCTCCTTCTGCTCAA]
308)>EL690583	[OPE-07]	442	[AGATGCAGTTGAAATCTT]	[CTGCATCTGTGATGCAAA]
309)>EL690609	[OPF-06]	235	[GGGAATTCCCTCCTTGTC]	[GAATTCCCCCGGACCAGC]
310) >EL690611	[OPG-14]	694	[GGATGAGATGACAAGCCT]	[TCTCATCCTCGTCAATGC]
311) >EL690651	[OPN-20]	236	[GGTGCTCCGCCTTAGGGGA]	[CGGAGCACCAGGTGGAGGG]
312) >EL690684	[OPN-08]	330	[ACCTCAGCATCAGCTGCT]	[AGCTGAGGTCGATAGGAAC]
313) >EL690691	[OPN-10]	395	[ACAACTGGGGATGGAACTGG]	[CCAGTTGTGATGGCCTCT]
314) >EL690710	[OPF-09]	319	[CCAAGCTTATCAGTAATT]	[AAGCTTGGAGAGATCGTC]
315) >EL690759	[OPL-03]	235	[CCAGCAGCGTCTGATCTT]	[GCTGCTGGTCCGGCGGAA]
316) >EL690879	[OPX-11]	227	[GGAGCCTCTGGTTCCCAA]	[CTGAGGCTCCCTCTCAAGGA]
317) >EL690896	[OPF-10]	658	[GGAAGCTTATTGCAGTTA]	[CAAGCTTCCAAATGGAACA]
318) >EL690905	[OPB-03]	650	[CATCCCCCATACTCTCGT]	[AGGGGGATGAGTCTGTTGG]
319) >EL690913	[OPC-16]	741	[CACACTCCTCTTCGTCTA]	[GGAGTGTGAGTCACTAGA]
320) >EL690938	[OPA-10]	887	[GTGATCGCTATACTACTA]	[GCGATCACTAGATTGTAC]
321) >EL690994	[OPR-19]	241	[CCTCCTCATCATGTCTACAA]	[TGAGGAGGAGGAGGAGGA]
322) >EL691025	[OPA-14]	301	[TCTGTGCTCAAGAGGGTG]	[AGCACAGACATAGCCCAC]
323) >EL691038	[OPM-01]	304	[GTTGGTGGTGGTGATGAT]	[CCACCAACAGCATTGAAG]
324) >EL691139	[OPI-13]	324	[CTGGGGCTTCTTTGAGAT]	[CAGCCCCAGGTTCTCACAC]
325) >EL691212	[OPI-02]	287	[GGAGGAGAAGAAGGAGAG]	[CTCTCCTCCACCTTCTCCA]
326) >EL691240	[OPI-17]	250	[GGTGGTGAAGCTCCCCCG]	[TCACCACCATCCCCATGA]
327) >EL691263	[OPN-08]	330	[ACCTCAGCATCAGCTGCT]	[AGCTGAGGTCGATAGGAAC]
328) >EL691265	[OPI-17]	250	[GGTGGTGAAGCTCCCCCG]	[TCACCACCATCCCCATGA]
329) >EL691291	[OPE-10]	315	[CACCAGGTGGAGACCATTC]	[ACCTGGTGATCTTGGTGA]
330) >EL691337	[OPD-04]	429	[TCTGGTGACCGCACACTG]	[TCACCAGAGAGCTTATGG]
331) >EL691341	[OPI-17]	250	[GGTGGTGAAGCTCCCCCG]	[TCACCACCATCCCCATGA]
332) >EL691422	[OPG-18]	408	[GGCTCATGTAAGGGGTCAT]	[ACATGAGCCCAAGTCTTCC]
333) >EL691490	[OPK-17]	446	[CCCAGCTGTTCATAAACTA]	[CAGCTGGGAAGATCAACC]
334) >EL691494	[OPJ-18]	235	[TGGTCGCACACTGGCTGA]	[TGCGACCATCCTCAAGTT]

335) >EL691626 [OPI-17] 228 [GGTGGTGATGGAGCTGCTGT] [CATCACCACCAACATGAAGT]
336) >EL691634 [OPI-16] 406 [TCTCCGCCGACAGGTTCA] [GGCGGAGATGGCGAGAGT]
337) >EL691669 [OPM-01] 304 [GTTGGTGGTGGTGATGAT] [CCACCAACAGCATTGAAG]
338) >EL691702 [OPK-04] 241 [CCGCCCAATTCGTTAAAG] [TTGGGCGGGTAGTTGGCC]
339) >EL691712 [OPB-04] 209 [GGACTGGAGATTACATGGT] [CTCCAGTCCCCATGCTCCT]
340) >EL691736 [OPL-18] 312 [ACCACCCACCTCCCCAGTAG] [TGGGTGGTTTTCATTTTG]
341) >EL691784 [OPC-15] 300 [GACGGATCTATCGAACCG] [CTGATCCGTCGATCAGGTGC]
342) >EL691869 [OPC-17] 353 [TTCCCCCCTTTAAAAAAA] [GGGGGGAAGCCCGTCTTC]
343) >EL691944 [OPN-12] 241 [CACAGACAACAATCTATA] [GTGTCTGTGCATGTGTGTG]
344) >EL691984 [OPS-03] 282 [CAGAGGTCCTATGGGCAGT] [GACCTCTGCTGGTGGTAC]
345) >EL692246 [OPF-04] 271 [GGTGATCATAAATATCGA] [TGATCACCTTGCAGCATT]
346) >EL692394 [OPC-06] 260 [GAACGGACTGGATGAATGG] [GTCCGTTCGGTCGTCGGT]
347) >EL692403 [OPC-17] 692 [TTCCCCCCCTCACAATCT] [GGGGGGAAGAAGGAGGAA]
348) >EL692404 [OPC-17] 376 [TTCCCCCCCTCTCTCTTC] [GGGGGGAAAAGAAGGGGA]
349) >EL692457 [OPZ-16] 417 [TCCCCATCTCCTCTTCTA] [GATGGGGAACAGGGACAC]
350) >EL692562 [OPI-17] 289 [GGTGGTGAGATTCAAGGA] [TCACCACCACACTGGTGT]
351) >EL692623 [OPC-17] 272 [TTCCCCCCACTCATCCTCA] [GGGGGGAAGGGGAGAGGA]
352) >EL692668 [OPB-18] 306 [CCACAGCATANGAATAAA] [TGCTGTGGGCGCTCTCTG]
353) >EL692671 [OPN-06] 957 [GAGACGCACAGCAGTGTCCA] [GTGCGTCTCTGTGCGTGAG]
354) >EL692689 [OPI-02] 266 [GGAGGAGACCCATGCCCT] [TCTCCTCCAACTTAAACA]
355) >EL692769 [OPI-02] 264 [GGAGGAGAAAGAGTTGTA] [TCTCCTCCTCCGCCTCTC]
356) >EL692780 [OPN-13] 233 [AGCGTCACTTAATCCGAAT] [GAGTGACGCTATTCGTGACT]
357) >EL692803 [OPI-02] 768 [GGAGGAGAATTCGCACAG] [TCTCCTCCTGAGCGGCCT]
358) >EL692845 [OPI-17] 367 [GGTGGTGACGAGGGAGTA] [TCACCACCATGGCCTCAA]
359) >EL692999 [OPZ-16] 220 [TCCCCATCTGCGAGATCT] [GATGGGGAGATCCACGGT]
360) >EL693037 [OPN-20] 238 [GGTGCTCCGCCTTAGGGGA] [CGGAGCACCCAGGTGGAGG]
361) >EL693054 [OPL-20] 235 [TGGTGGACTTGCATGGGA] [GGTCCACCATGGTTGTGTA]
362) >EL693306 [OPN-12] 206 [CACAGACAAGACATCCAT] [TGTCTGTGTACTAAGTCC]
363) >EL693317 [OPL-11] 1902 [ACGATGAGGAATGAGAGG] [CTCATCGTTCTGTCTGGC]
364) >EL693363 [OPZ-07] 250 [CCAGGAGGTCTGATGAGC] [CCTCCTGGCCACTCCAAA]
365) >EL693364 [OPZ-07] 253 [CCAGGAGGTCTGATGAGC] [CCTCCTGGGCCACGTCCG]
366) >EL693485 [OPQ-19] 549 [CCCCCTATGCATGGAGTG] [ATAGGGGGAGTAACTCTC]
367) >EL693512 [OPC-14] 661 [TGCGTGCTGCCGTGTCGC] [AGCACGCACACGCNACAG]
368) >EL693558 [OPL-10] 261 [TGGGAGATATGCCTAGAG] [CATCTCCCACAAGTTCAAC]
369) >EL693568 [OPE-09] 350 [CTTCACCCTCATCGTCCT] [CGGGTGAAGTAGATGTAGC]
370) >EL693616 [OPN-20] 236 [GGTGCTCCGCCTTAGGGGA] [CGGAGCACCAGGTGGAGGG]
371) >EL693620 [OPB-03] 234 [CATCCCCCAGACCAGCAG] [GGGGGATGCCCTCCTTGT]
372) >EL693663 [OPB-13] 237 [TTCCCCCGTGACAGCAAA] [CGGGGGAATTCCCTCCTT]
373) >EL693671 [OPK-02] 620 [GTCTCCGCGCAACAACAC] [GCGGAGACAGTTTATGTG]
374) >EL693812 [OPA-05] 394 [AGGGGTCTTCAATGGGTCG] [AAGACCCCTGCGTAAAAGT]
375) >EL693834 [OPC-16] 559 [CACACTCCTCCTGCCACC] [GGAGTGTGCGGCTGCAGG]
376) >EL693896 [OPG-14] 1062 [GGATGAGATGTAGTGTAA] [TCTCATCCACAGACTCAT]
377) >EL693902 [OPE-07] 469 [AGATGCAGGATGTGGAGG] [CTGCATCTTGACAACACT]
378) >EL693954 [OPN-06] 594 [GAGACGCATTCTACTCAA] [TGCGTCTCAGTCGGGTGG]
379) >EL693955 [OPL-18] 509 [ACCACCCAACTCATTGCC] [TGGGTGGTATGAATTGTA]
380) >EL693996 [OPA-10] 260 [GTGATCGCGCATGGCGGT] [GCGATCACGTCACACGTC]
381) >EL693999 [OPP-14] 1265 [CCAGCCGAGCCGCGGACA] [TCGGCTGGGCATAAAGCG]
382) >EL694003 [OPN-12] 444 [CACAGACAGGACCAAACA] [TGTCTGTGAGGTTGTGTG]
383) >EL694021 [OPI-17] 1522 [GGTGGTGACCCACTTTTC] [TCACCACCAGATNACATA]
384) >EL694050 [OPE-15] 1419 [ACGCACAAACATNTAGGA] [TTGTGCGTGCGTGCAGCC]
385) >EL694066 [OPE-15] 231 [ACGCACAACGACAGAAAGA] [TTGTGCGTGTGTGGGGGT]
386) >EL694082 [OPF-17] 310 [AACCCGGGACATTGCTTCG] [CCCGGGTTAAATTGCCGG]
387) >EL694250 [OPK-15] 249 [CTCCTGCCTGCCTCAACT] [TGGCAGGAGCAGAGAGTGC]
388) >EL694327 [OPL-18] 295 [ACCACCCAGGGTGTCACC] [GTGGGTGGTGTGGGAGAGG]
389) >EL694627 [OPN-08] 250 [ACCTCAGCAAAAGCCCAG] [GCTGAGGTGGGACCCATC]
390) >EL694717 [OPQ-02] 500 [TCTGTCGGTGTGGTTTGAT] [CCGACAGAAGTGCATCCA]

391) >EL694827 [OPB-14] 336 [TCCGCTCTCCTCCATCTC] [AGAGCGGAGACGAGGCTG]
392) >EL694955 [OPZ-07] 516 [CCAGGAGGACTCAACCCGCC] [CCTCCTGGCAGAACCTCA]
393) >EL694961 [OPF-14] 212 [TGCTGCAGTTGCCTGCCC] [CTGCAGCAGAACTGCCCG]
394) >EL694967 [OPX-19] 689 [TGGCAAGGGGATTGAAGA] [CCTTGCCAATGGTGAAGA]
395) >EL695053 [OPB-13] 235 [TTCCCCCGGACCAGCAGA] [CGGGGGAATTCCCTCCTT]
396) >EL695133 [OPZ-07] 516 [CCAGGAGGACTCAACCCGCC] [CCTCCTGGCAGAACCTCA]
397) >EL695166 [OPZ-07] 516 [CCAGGAGGACTCAACCCGCC] [CCTCCTGGCAGAACCTCA]
398) >EL695246 [OPL-03] 347 [CCAGCAGCTATGAACGCAT] [GCTGCTGGAGAGAGTCTT]
399) >EL695306 [OPY-15] 359 [AGTCGCCCCCGATGTCTC] [GGGCGACTTCCGCCTTCC]
400) >EL695332 [OPX-08] 304 [CAGGGGTGGCCGGCAATGG] [CACCCCTGCATGCAGCAT]
401) >EL695346 [OPG-20] 327 [TCTCCCTCCTGCTCCTGC] [GAGGGAGAGCTGCAGTAC]
402) >EL695500 [OPM-18] 336 [CACCATCCCTCACATAAC] [GGATGGTGTGTGTAAAAG]
403) >EL930251 [OPI-16] 204 [TCTCCGCCTCTGAGCTCC] [GGCGGAGAGGACGGGGCT]
404) >EL930269 [OPQ-16] 357 [AGTGCAGCTGTATCTCAA] [GCTGCACTTGAGATTGTG]
405) >EL930296 [OPJ-05] 295 [CTCCATGGATGAGGATCA] [CCATGGAGTCAACCCCAC]
406) >EL930309 [OPF-04] 434 [GGTGATCACCACCTATGA] [TGATCACCCAAAACTTAN]
407) >EL930316 [OPB-03] 241 [CATCCCCCCGGACCAGCA] [GGGGGATGCCCTCCCTNG]
408) >EL930342 [OPD-09] 299 [CTCTGGAGCATGATTACA] [CTCCAGAGAACCCCCACT]
409) >EL930358 [OPI-02] 287 [GGAGGAGAAGAAGGAGAG] [CTCTCCTCCACCTTCTCCA]
410) >EL930421 [OPI-02] 471 [GGAGGAGAGAGTGGGTTAG] [TCTCCTCCAGGTCCCAGA]
411) >EL930449 [OPA-10] 220 [GTGATCGCCAACGGTCCG] [GCGATCACGGCGGTATCG]
412) >EL930467 [OPQ-17] 308 [GAAGCCCTTGTCGGTCCTCT] [AAGGGCTTCCCCTTGAGCA]
413) >EL930518 [OPI-16] 271 [TCTCCGCCTCCGCCGCCA] [GGCGGAGAAGAGCTGGCC]
414) >EL930565 [OPE-07] 241 [AGATGCAGAGATGGATGA] [CTGCATCTGAATTGTGAA]
415) >EL930589 [OPI-02] 431 [GGAGGAGAGAGAGAGAGAG] [CCTCTCCTCCTCCACCGTCG]
416) >ES273657 [OPB-03] 235 [CATCCCCCCGGACCAGCA] [GGGGGATGCCCTCCTTGT]
417) >ES273659 [OPD-02] 534 [GGACCCAATCTTCTTCTA] [TTGGGTCCCTGTTCAACC]
418) >ES273678 [OPA-13] 327 [CAGCACCCGAATCTTGTG] [GGGTGCTGATGTGGGTGT]
419) >ES273686 [OPI-17] 356 [GGTGGTGATGTTGAACTTTA] [CATCACCACCATTTCTTRAT]
420) >ES273689 [OPI-02] 431 [GGAGGAGAGAGAGAGAGAG] [CCTCTCCTCCTCCACCGTCG]
421) >ES273720 [OPF-14] 346 [TGCTGCAGGAATCAAGTTA] [CTGCAGCACCTAGAACTG]
422) >ES273721 [OPB-03] 235 [CATCCCCCCGGACCAGCA] [GGGGGATGCCCTCCTTGT]
423) >ES273743 [OPP-03] 235 [CTGATACGATTGATAATG] [CGTATCAGAGCTCTCAAC]
424) >ES273749 [OPP-03] 235 [CTGATACGATTGATAATG] [CGTATCAGAGCTCTCAAC]
425) >ES273811 [OPP-13] 378 [GGAGTGCCTCGATTGTCATT] [AGGCACTCCTCCTGTGTGC]
426) >ES273836 [OPR-19] 240 [CCTCCTCATCCATGAGGGCG]
[GATGAGGAGGGCCCCGCTGG]
427) >ES273871 [OPB-03] 235 [CATCCCCCCGGACCAGCA] [GGGGGATGCCCTCCTTGT]
428) >ES273877 [OPB-03] 235 [CATCCCCCCAGACCAGCA] [GGGGGATGCCCTCCTTGT]
429) >ES273881 [OPI-02] 212 [GGAGGAGAACGTGTACAT] [CTCTCCTCCTTCTGCTCGA]
430) >ES323722 [OPB-03] 463 [CATCCCCCCTGACCAGCA] [GGGGGATGCCCTCCTTAT]
431) >ES323762 [OPG-20] 563 [TCTCCCTCAACCATGGCAT] [GAGGGAGAGGGGTGCATC]
432) >ES323813 [OPM-08] 332 [TCTGTTCCNTTCTTTCTT] [GGAACAGAGCGAGGAGAG]
433) >ES323817 [OPK-03] 340 [CCAGCTTAAAGCTAAAAG] [TAAGCTGGTGCTATATCA]
434) >ES323859 [OPX-08] 236 [CAGGGGTGGCATGCAGATC] [CCACCCCTGAGCCGCAGAA]
435) >ES323868 [OPP-03] 235 [CTGATACGATTGATAATG] [CGTATCAGAGCTCTCAAC]
436) >ES323874 [OPZ-20] 235 [ACTTTGGCTGACTACAAC] [GCCAAAGTGCGACCATCC]
437) >ES323893 [OPJ-15] 228 [TGTAGCAGGATGCAAGCTT] [CCTGCTACACCTACTAGCC]
438) >ES324003 [OPL-07] 372 [AGGCGGGAGACGGGGACG] [TCCCGCCTCTCCCCCCAC]
439) >ES324037 [OPB-03] 235 [CATCCCCCCGGACCAGCA] [GGGGGATGCCCTCCTTGT]
440) >ES324063 [OPB-03] 235 [CATCCCCCCAGACCAGCA] [GGGGGATGCCCTCCTTGT]
441) >ES370553 [OPQ-17] 265 [GAAGCCCTGTTCGTGCAC] [AGGCTTCTGCTTATGCT]
442) >ES370561 [OPM-18] 272 [CACCATCCCCGGCGCCCG] [GGATGGTGTAGNTGAGGC]
443) >ES370595 [OPI-02] 287 [GGAGGAGAAGAAGGAGAG] [CTCTCCTCCACCTTCTCCA]
444) >ES370622 [OPB-03] 234 [CATCCCCCCGGACCAGCA] [GGGGGATGCCCTCCTTGT]
445) >ES370656 [OPP-13] 379 [GGAGTGCCTCGATTGTCATT] [AGGCACTCCTCCTGTGTTG]
446) >ES370729 [OPL-03] 235 [CCAGCAGCGTCTGATCTT] [GCTGCTGGTCCGGCGGAA]

447)>ES370811	[OPB-03]	235	[CATCCCCCCAGACCAGCA]	[GGGGGATGCCCTCCTTGT]
448)>ES370825	[OPI-02]	212	[GGAGGAGAACGTGTACAT]	[CTCTCCTCCTTCTGCTCGA]
449)>ES370900	[OPB-03]	234	[CATCCCCCCGGACCAGCA]	[GGGGGATGCCNTCCTTGT]
450)>ES370908	[OPF-03]	482	[CCTGATCAATCGCTGGAG]	[GTGATCAGGCTGATGAGCG]
451) >ES414425	[OPX-12]	336	[TCGCCAGCTGGGAGGATG]	[TGGCTGGCGACGTTCTCGCG]
452) >ES414428	[OPB-03]	393	[CATCCCCCGCCTCAAAAA]	[GGGGGATGCTGCCCGTCT]
453)>ES414488	[OPA-13]	327	[CAGCACCCGAATCTTGTG]	[GGGTGCTGATGTGGGTGT]
454)>ES414497	[OPG-20]	328	[TCTCCCTCACCTTCATCAC]	[GAGGGAGAGCAGCAATAG]
455)>ES414548	[OPI-16]	605	[TCTCCGCCCACAGTTCCTC]	[GGCGGAGATGAGCTGCTG]
456)>ES414552	[OPR-19]	206	[CCTCCTCAGCCACCGCCG]	[TGAGGAGGAGAACGAGGG]
457)>ES414596	[OPL-03]	235	[CCAGCAGCGTCTGATCTT]	[GCTGCTGGTCCGGCGGAA]
458)>ES414649	[OPJ-11]	326	[ACTCCTGCTCAAGATAAA]	[GCAGGAGTTGGCCACAGC]
459)>ES414732	[OPZ-16]	418	[TCCCCATCTCCCCACTAT]	[GATGGGGATGAGAGAATC]
460)>ES414739	[OPI-02]	460	[GGAGGAGAGGACGAAGAAGA]	[TCTCCTCCACTTGAGTCA]
461) >ES414773	[OPB-06]	457	[TGCTCTGCGACGCCGAGG]	[GCAGAGCAGTGGNATCCC]
462)>EY396184	[OPZ-05]	406	[TCCCATGCCTCCAATGTC]	[GCATGGGATGCATGCCAT]
463)>EY396208	[OPM-01]	304	[GTTGGTGGAGGTGATGAT]	[CCACCAACAGCATTGAAG]
464)>EY396328	[OPI-17]	367	[GGTGGTGACGAGGGAGTA]	[TCACCACCATGGCCTCAA]
465)>EY396489	[OPP-13]	254	[GGAGTGCCACTGCGTGCC]	[GGCACTCCAAAAACAACT]
466)>EY396578	[OPP-13]	254	[GGAGTGCCACTGCGTGCC]	[GGCACTCCAAAAACAACT]
467)>EY396802	[OPB-17]	303	[AGGGAACGTTCCAAAAAC]	[CGTTCCCTTGCCCTTCTT]
468)>EY397142	[OPJ-15]	340	[TGTAGCAGTACCAGCAAA]	[CTGCTACATACTTATCTA]
469)>EY398259	[OPX-05]	217	[CCTTTCCCAGCGCAGCAG]	[GGGAAAGGCATGGACTTC]
470)>EY398616	[OPE-10]	397	[CACCAGGTGCAATTACTCC]	[ACCTGGTGATGAATTAAT]
471) >EY398667	[OPI-02]	311	[GGAGGAGAGGGCATTTTCTG]	[CTCTCCTCCTCAAATTGTT]
472) >EY399503	[OPA-13]	435	[CAGCACCCTATGCCACTG]	[GGGTGCTGGCCTGCTGCT]
473) >EY399570	[OPF-13]	340	[GGCTGCAGAGATACTTTCA]	[CTGCAGCCATGGGGACCG]
474) >EY399637	[OPL-09]	247	[TGCGAGAGGGGGAATCCC]	[CTCTCGCAGCAGCTCCAA]
475) >EY400003	[OPE-07]	371	[AGATGCAGTTTCGAATTT]	[CTGCATCTGCCTCCTGAA]
476)>EY400240	[OPF-09]	379	[CCAAGCTTCCACCAATTTTT]	[AAGCTTGGGTCTGCTCGG]
477) >EY400513	[OPE-07]	255	[AGATGCAGGCTCAAGAAA]	[CTGCATCTTGGCAGAGCT]
478)>EY400555	[OPJ-15]	340	[TGTAGCAGTACCAGCAAA]	[CTGCTACATACTTATCCA]
479)>EY400580	[OPF-13]	340	[GGCTGCAGAGATACTTTCA]	[CTGCAGCCATGGGGACCG]
480)>EY400593	[OPL-09]	247	[TGCGAGAGGGGGAATTCC]	[CTCTCGCAGTAGCTCCAA]
481) >EY401002	[OPE-07]	314	[AGATGCAGCTGTTGGCATT]	[CTGCATCTGCAATTAGCA]
482)>EY401213	[OPZ-04]	286	[AGGCTGTGGTAAGGCCTT]	[CACAGCCTTCATATGGAC]
483)>EY401537	[OPF-09]	379	[CCAAGCTTCCACCAATTTTT]	[AAGCTTGGGTCTGCTCGG]
484)>EY401560	[OPF-03]	310	[CCTGATCAAGGAAGTGGA]	[TGATCAGGTGTTTCCCCA]
485)>EY401593	[OPI-09]	433	[TGGAGAGCACCCGCTGAGC]	[GCTCTCCATCATGTTTCT]
486)>EY402047	[OPF-04]	508	[GGTGATCATGCTGGTGTT]	[TGATCACCGGAAGCAGTG]
487)>EY402331	[OPE-03]	404	[CCAGATGCAGTGGTTCTCC]	[TGCATCTGGAGGTCTGGCC]
488)>EY402355	[OPI-09]	433	[TGGAGAGCACCCGCTGAGC]	[GCTCTCCATCATGTTTCT]
489)>EY402472	[OPF-13]	340	[GGCTGCAGAGATACTTTCA]	[CTGCAGCCATGGGGACCG]
490)>EY402492	[OPI-17]	433	[GGTGGTGAAGATGCAGTA]	[TCACCACCACTCGCGAAA]
491) >EY402552	[OPG-18]	407	[GGCTCATGTAAGGGGTCAT]	[ACATGAGCCCAAGTCTTCC]
492)>EY402658	[OPF-06]	351	[GGGAATTCCTATCAATAG]	[GAATTCCCCCTCTGAGAA]
493)>EY403057	[OPZ-17]	330	[CCTTCCCAACAGGCAAAG]	[TGGGAAGGGAAGAATGCC]
494)>EY403263	[OPF-10]	607	[GGAAGCTTACATGGATAT]	[AAGCTTCCATCAAGAGTA]
495)>EY403507	[OPJ-15]	340	[TGTAGCAGTACCAGCAAA]	[CTGCTACATACTTATCCA]
496)>EY403695	[OPF-14]	201	[TGCTGCAGTTCCAACGTT]	[CTGCAGCAGGGAGGATCA]
497)>EY403747	[OPI-02]	297	[GGAGGAGAGAGAGGAAAAG]	[TCTCCTCCTCTCTTGCAT]
498)>EY404142	[OPI-17]	287	[GGTGGTGATTCAAGGAGAT]	[TCACCACCACACTGGTGT]
499)>EY404157	[OPP-13]	254	[GGAGTGCCACTGCGTGCC]	[GGCACTCCAAAAACAACT]
500)>EY404781	[OPL-10]	570	[TGGGAGATTGTGAAGAGA]	[ATCTCCCAGAAAACTTAA]
501) >EY405716	[OPF-14]	319	[TGCTGCAGGAAGAATCAAG]	[CTGCAGCAGCAGGAAGAT]
502)>EY405887	[OPY-12]	237	[AAGCCTGCTTCATAAGCT]	[GCAGGCTTGTAGCGGACA]
503)>EY406341	[OPY-12]	237	[AAGCCTGCTTCATAAGCT]	[GCAGGCTTGTAGCGGACA]

504)>EY406875	[OPG-18] 264	[GGCTCATGTTGGGTCGACA]	[ACATGAGCCTCACTTCCAC]
505)>EY407695	[OPL-20] 235	[TGGTGGACTTGCATGGGA]	[GGTCCACCATGGTTGTGTA]
506)>EY407868	[OPL-03] 268	[CCAGCAGCAACAGCCTGT]	[GCTGCTGGGGTTTCTGTA]
507)>EY407993	[OPC-04] 202	[CCGCATCTTTGTTTCACT]	[AGATGCGGCGATCCGGTC]
508)>EY408031	[OPY-02] 259	[CATCGCCGGCAATGGGAG]	[CGGCGATGGCTCGCCGTC]
509)>EY408183	[OPL-03] 268	[CCAGCAGCAACAGCCTGT]	[GCTGCTGGGGTTTCTGTA]
510) >EY408246	[OPR-19] 412	[CCTCCTCAAAACCCTAAC]	[ATGAGGAGGCGGGGCTCAC]
511) >EY408272	[OPI-12] 274	[AGAGGGCAAAATTGTTCT]	[TGCCCTCTTACTTAAGGT]
512) >EY408390	[OPL-20] 302	[TGGTGGACTCCACCCACG]	[GGTCCACCATGTTGAGTTG]
513) >EY408521	[OPL-03] 235	[CCAGCAGCGTCTGATCTT]	[GCTGCTGGTCCGGCGGAA]
514) >EY408526	[OPJ-18] 235	[TGGTCGCACCTTGGCAGA]	[TGCGACCATCCTCAAGCT]
515) >EY408585	[OPE-18] 436	[GGACTGCATGGTCATTTG]	[TGCAGTCCCACCAGATCC]
516) >EY408592	[OPF-13] 223	[GGCTGCAGCTGATGATGT]	[CTGCAGCCTCCTCTCTTT]
517) >EY408627	[OPR-19] 560	[CCTCCTCATCATGCCTACAA]	[GATGAGGAGGAGGTGGTGAT]
518) >EY408659	[OPQ-16] 357	[AGTGCAGCTGTATCTCAA]	[GCTGCACTTGAGATTGTG]
519) >EY408714	[OPE-09] 355	[CTTCACCCACCACAACGA]	[GGGTGAAGTAGGCGCCAT]
520)>EY408741	[OPB-03] 235	[CATCCCCCCAGACCAGCA]	[GGGGGATGCCCTCCTTGT]
521) >EY408793	[OPL-03] 235	[CCAGCAGCGTCTGATCTT]	[GCTGCTGGTCCGGCGGAA]
522) >EY408853	[OPI-02] 235	[GGAGGAGAAGGGAGGGGG]	[CCTCTCCTCCGATCCGGAAC]
523) >EY408913	[OPK-15] 313	[CTCCTGCCGCGAGGGCAT]	[GGCAGGAGGTGCTGCAGC]
524) >EY408951	[OPI-09] 433	[TGGAGAGCACCCGCTGAGC]	[GCTCTCCATCATGTTTCT]
525) >EY409146	[OPL-03] 235	[CCAGCAGCGTCTGATCTT]	[GCTGCTGGTCCGGCGGAA]
526)>EY409169	[OPE-18] 436	[GGACTGCATGGTCATTTG]	[TGCAGTCCCACCAGATCC]
527) >EY409202	[OPZ-20] 235	[ACTTTGGCTGACTACAAC]	[GCCAAAGTGCGACCATCC]
528)>EY409207	[OPE-07] 597	[AGATGCAGCCACAGCAGAGC]	[CTGCATCTGAGGGACATC]
529)>EY409248	[OPN-08]366	[ACCTCAGCATCAGCTGCT]	[AGCTGAGGTCGATAGGAAT]
530)>EY409270	[OPB-06] 245	[TGCTCTGCGGAAAATGCT]	[GCAGAGCATAAGCTTCCT]
531) >EY409335	[OPI-02] 296	[GGAGGAGATGTACGCGGA]	[TCTCCTCCAGATATAATG]
532)>EY409432	[OPL-20] 302	[TGGTGGACTCCACCCACG]	[GGTCCACCATGTTGAGTTG]
533)>EY409444	[OPR-19] 477	[CCTCCTCATCATGCCTACAA]	[GATGAGGAGGATGTGGTGAT]
534)>EY409481	[OPL-08] 245	[AGCAGGTGACAGCGCAGG]	[CACCTGCTTTCAGAATCG]
535)>EY409771	[OPF-04] 271	[GGTGATCATAAATATCGA]	[TGATCACCTTGCAGCATT]
536)>EY409930	[OPJ-18] 235	[TGGTCGCACACTGGCTGA]	[TGCGACCATCCTCAAGTT]
537)>EY409976	[OPN-20]236	[GGTGCTCCGCCTTAGGGGA]	[CGGAGCACCAGGTGGAGGG]
538)>EY410007	[OPI-17] 286	[GGTGGTGATTCAGGAGATG]	[TCACCACCACACTGGTGT]
539)>EY410059	[OPJ-15] 340	[TGTAGCAGTACCAGCAAA]	[CTGCTACATACTTATCTA]
540)>EY410089	[OPL-15] 427	[AAGAGAGGATGGCCAAGT]	[CCTCTCTTCCTCGACCTC]
541) >EY410238	[OPE-18] 436	[GGACTGCATGGTCATTTG]	[TGCAGTCCCACCAGATCC]
542)>EY410239	[OPE-18] 436	[GGACTGCATGGTCATTTG]	[TGCAGTCCCACCAGATCC]
543)>EY410319	[OPB-08] 419	[GTCCACACTCTCGATTCC]	[GTGTGGACCGCGACGTCC]
544)>EY410345	[OPN-08]366	[ACCTCAGCATCAGCTGCT]	[AGCTGAGGTCGATAGGAAT]
545)>EY410402	[OPK-15] 313	[CTCCTGCCGCGAGGGTAT]	[GGCAGGAGGTGCTGCAGC]
546)>EY410445	[OPI-17] 287	[GGTGGTGATTCAAGGAGAT]	[TCACCACCACACTGGTGT]
547)>EY410477	[OPL-20] 302	[TGGTGGACTCCACCCACG]	[GGTCCACCATGTTGAGTTG]
548)>EY410521	[OPE-18] 437	[GGACTGCATGGTCATTTG]	[TGCAGTCCCACCAGATCC]
549)>EY410536	[OPR-20] 215	[ACGGCAAGGGCGGCCTTTC]	[CTTGCCGTGATTTCACGC]
550)>EY410710	[OPI-13] 324	[CTGGGGCTTCTTTGAGAT]	[CAGCCCCAGGTTCTCACAC]
551) >EY410735	[OPR-10] 533	[CCATTCCCTCTTTCCTAA]	[GGGGAATGGCGAGGTGGGG]
552)>EY410745	[OPI-02] 235	[GGAGGAGAAGGGAGGGGG]	[CCTCTCCTCCGATCCGGAAC]
553)>EY410939	[OPE-18] 436	[GGACTGCATGGTCATTTG]	[TGCAGTCCCACCAGATCC]
554)>EY410948	[OPN-08]366	[ACCTCAGCATCAGCTGCT]	[AGCTGAGGTCGATAGGAAT]
555)>EY410955	[OPF-14] 201	[TGCTGCAGTTCCAACGTT]	[CTGCAGCAGGGAGGATCA]
556)>EY410964	[OPE-18] 436	[GGACTGCATGGTCATTTG]	[TGCAGTCCCACCAGATCC]
557)>EY410998	[OPX-05] 309	[CCTTTCCCTATGTCTTTTG]	[GGGAAAGGAATCCATCCA]
558)>EY411072	[OPI-17] 289	[GGTGGTGAGATTCAAGGA]	[TCACCACCACACTGGTGT]
559)>EY411194	[OPL-03] 235	[CCAGCAGCGTCTGATCTT]	[GCTGCTGGTCCGGCGGAA]
560)>EY411231	[OPN-20] 413	[GGTGCTCCTATTGAAGAT]	[CGGAGCACCAGTCACAAAT]

561) >EY411277 [OPY-19] 203 [TGAGGGTCCTCGGCCCCCA] [GACCCTCATTTTTTCGAG]
562) >EY411596 [OPI-02] 212 [GGAGGAGAACGTGTACAT] [CTCTCCTCCTTGAGCTCGA]
563) >EY411649 [OPN-08]366 [ACCTCAGCATCAGCTGCT] [AGCTGAGGTCGATAGGAAT]
564) >EY411746 [OPI-02] 220 [GGAGGAGAAGAAAGGTTT] [TCTCCTCCCCTCCATTCC]
565) >EY411756 [OPZ-07] 250 [CCAGGAGGTCTCAGGAGC] [CCTCCTGGCAACTCCAAA]
566) >EY411790 [OPL-03] 235 [CCAGCAGCGTCTGATCTT] [GCTGCTGGTCCGGCGGAA]
567) >EY411834 [OPR-19] 412 [CCTCCTCAAAACCCTAAC] [ATGAGGAGGCGGGGCTCAC]
568) >EY411857 [OPR-19] 450 [CCTCCTCATGTTTACTACT] [GATGAGGAGGAGGTGGTGAT]
569) >EY411908 [OPN-08]366 [ACCTCAGCATCAGCTGCT] [AGCTGAGGTCGATAGGAAT]
570) >EY411946 [OPN-08]366 [ACCTCAGCATCAGCTGCT] [AGCTGAGGTCGATAGGAAT]
571) >EY411977 [OPI-17] 287 [GGTGGTGATTCAAGGAGAT][TCACCACCACACTGGTGT]
572) >EY412036 [OPY-12] 423 [AAGCCTGCCAAAGGTGGA] [GCAGGCTTGCTGAAAGAT]
573) >EY412080 [OPI-17] 325 [GGTGGTGAGATCCTCGGA] [TCACCACCCTCACACACC]
574) >EY412100 [OPE-20] 242 [AACGGTGAATAAGAAGGA] [GTCACCGTTATGGTATCAT]
575) >EY412161 [OPR-07] 201 [ACTGGCCTGTTGTTGCTTT] [AGGCCAGTAGCTTCTGGT]
576) >EY412284 [OPL-20] 302 [TGGTGGACTCCACCCACG] [GGTCCACCATGTTGAGTTG]
577) >EY412338 [OPX-11] 321 [GGAGCCTCAGTCTCTGTTGG] [CTGAGGCTCCTACTCTTACT]
578) >EY412534 [OPE-18] 436 [GGACTGCATGGTCATTTG] [TGCAGTCCCACCAGATCC]
579) >EY412647 [OPN-08]366 [ACCTCAGCATCAGCTGCT] [AGCTGAGGTCGATAGGAAT]
580) >EY412721 [OPL-10] 508 [TGGGAGATTACTCATATA] [CATCTCCCAACCCTGGGAC]
581) >EY412740 [OPI-02] 277 [GGAGGAGACCGGCTTTGT] [TCTCCTCCTCGAGCTCGA]
582) >EY412905 [OPF-14] 310 [TGCTGCAGCTTTGGTACT] [CCTGCAGCATCCGCCGCGA]
583) >EY412958 [OPE-18] 436 [GGACTGCATGGTCATTTG] [TGCAGTCCCACCAGATCC]
584) >EY413024 [OPI-17] 287 [GGTGGTGATTCAAGGAGAT][TCACCACCACACTGGTGT]
585) >EY413048 [OPQ-02]500 [TCTGTCGGTGTGGTTTGAT] [CCGACAGAAGTGCATCCA]
586) >EY413225 [OPD-17] 269 [TTTCCCACACATAATAAC] [GTGGGAAAAAGGTCCCCA]
587) >EY413249 [OPE-18] 436 [GGACTGCATGGTCATTTG] [TGCAGTCCCACCAGATCC]
588) >EY413253 [OPQ-02]500 [TCTGTCGGTGTGGTTTGAT] [CCGACAGAAGTGCATCCA]
589) >EY413439 [OPB-10] 486 [CTGCTGGGTAGTTACTTC] [GTCCCAGCAGACGGCCAACA]
590) >EY413491 [OPI-17] 287 [GGTGGTGATTCAAGGAGAT][TCACCACCACACTGGTGT]
591) >EY413517 [OPL-03] 235 [CCAGCAGCGTCTGATCTT] [GCTGCTGGTCCGGCGGAA]
592) >EY413546 [OPI-17] 289 [GGTGGTGAGATTCAAGGA] [TCACCACCACACTGGTGT]
593) >EY413594 [OPI-02] 205 [GGAGGAGACCCAGATTCT] [TCTCCTCCCGATATATGC]
594) >EY413619 [OPE-18] 436 [GGACTGCATGGTCATTTG] [TGCAGTCCCACCAGATCC]
595) >GH635938 [OPF-09] 319 [CCAAGCTTATCAGTTATT] [AAGCTTGGAGAGATCGTC]
596) >GH635980 [OPR-19] 775 [CCTCCTCAAAATGTGCAA] [TGAGGAGGCTTTAGAAAA]
597) >GH636097 [OPZ-20] 470 [ACTTTGGCCAGCAGTTGC] [GCCAAAGTGGTGGGCATG]
598) >GH636115 [OPP-19] 222 [GGGAAGGACTACGGCAGGC] [TCCTTCCCCACGAGCTGT]
599) >GH636200 [OPF-17] 282 [AACCCGGGGGGTTTAAAC] [CCCGGGTTTTTTTGATTT]
600) >GH636320 [OPN-08]252 [ACCTCAGCATCCTGCTCT] [AGCTGAGGTATGCTCTTTT]
601) >GH636362 [OPC-17] 331 [TTCCCCCCCAAAAAAAAA] [GGGGGGAACCCAAATTTT]
602) >GH636388 [OPC-17] 298 [TTCCCCCCGGNGNNNNGA] [TGGGGGGAAGGGGAAATTT]
603) >GH636478 [OPC-17] 240 [TTCCCCCCCCGGTTTAAA] [TGGGGGGAAACCGGGGATT]
604) >GH636518 [OPC-17] 203 [TTCCCCCCCAAAAAAGCC] [TGGGGGGAATTGGGGCNNN]
605) >GH636519 [OPX-05] 380 [CCTTTCCCCCTTTTTTTC] [GGGAAAGGGTGGGTCATT]
606) >GH636646 [OPC-02] 359 [GTGAGGCGCCCCAACAAT] [CGCCTCACACCTTTCATT]
607) >GH636694 [OPM-08] 222 [TCTGTTCCATTTTCCGCA] [GGAACAGAGATGTGGCCG]
608) >GH636748 [OPP-03] 235 [CTGATACGATTGATAATG] [CGTATCAGAGCNNNCAAC]
609) >GH636752 [OPB-13] 453 [TTCCCCCGGGAAAAAAAA] [CGGGGGAAGTTTTTTACC]
610) >GH636785 [OPF-17] 230 [AACCCGGGGGGGATCGGG] [CCCGGGTTTTTTCCGGCC]
611) >GH636799 [OPC-17] 321 [TTCCCCCCGGGAAAAATT] [GGGGGGAATTTATTTTGG]
612) >GH636920 [OPJ-07] 496 [CCTCTCGATCATGTTATC] [TCGAGAGGNNNGAGAAAG]
613) >GH636995 [OPI-02] 239 [GGAGGAGAGCCTCATCCTA] [TCTCCTCCTGTTCCTANN]
614) >GH637007 [OPC-17] 338 [TTCCCCCCTTTTTCTTTT] [GGGGGGAANNNTTGGGAA]
615) >GH637018 [OPC-17] 341 [TTCCCCCCAAAAAATTTTT] [GGGGGGAAAAGGGGGTTT]
616) >GH637093 [OPC-17] 599 [TTCCCCCCCCAAAAAAAT] [TGGGGGGAATTGGGCCNNN]
617) >GH637184 [OPK-04] 297 [CCGCCCAAAAATTAACCCA] [TTGGGCGGGGGGGTCACC]

618) >GH637218	[OPB-13]	356	[TTCCCCCGGGCCAAGAAC]	[CGGGGGAAAAAAAATTTT]
619) >GH637247	[OPC-17]	384	[TTCCCCCCCAGAAAAAAA]	[GGGGGGAAACGGGTTGGG]
620)>GH637286	[OPC-17]	387	[TTCCCCCCAGAGGCCCGGGC]	[TGGGGGGAATTTGGCCCCN]
621) >GH637290	[OPB-15]	241	[GGAGGGTGGAATTGGGAG]	[CACCCTCCCATCGTAGCC]
622) >GH637317	[OPC-17]	246	[TTCCCCCCCCCCAAAATT]	[GGGGGGAAAAATTTTTTC]
623) >GH637462	[OPE-07]	206	[AGATGCAGTTCACCCCAG]	[CTGCATCTTTGCCATTTG]
624) >GH637474	[OPC-17]	242	[TTCCCCCCTGGAAAAAAA]	[GGGGGGAATTTTTTTTGA]
625) >GH637585	[OPC-17]	264	[TTCCCCCCCCAAAAAAGA]	[GGGGGGAAAAATTGCCCC]
626)>GH637587	[OPC-17]	239	[TTCCCCCCCCTCCAAAAC]	[GGGGGGAAACCCCCGCCC]
627) >GH637627	[OPC-17]	281	[TTCCCCCCAAAAACCAGTT]	[GGGGGGAAAAATTTAAAT]
628)>GH637639	[OPP-19]	222	[GGGAAGGACTATGGCAGGC]	[TCCTTCCCCATGAGCTGT]
629)>GH637757	[OPR-19]	391	[CCTCCTCAAGATCAGCCT]	[TGAGGAGGTGCACGTGCT]
630)>GR930411	[OPC-17]	200	[TTCCCCCCCCACGGGGGG]	[GGGGGGAATTTGGGCGGA]
631) >GT119125	[OPY-19]	235	[TGAGGGTCTTCACAAAAA]	[GACCCTCACTGGAAAGAC]
632) >GT119167	[OPX-11]	227	[GGAGCCTCTGGTTCCCAA]	[CTGAGGCTCCCTCTCAAGGA]
633) >GT119196	[OPI-02]	222	[GGAGGAGAGGTAGCGGTCGG]	[TCTCCTCCTTCTTCCCTC]
634) >GT119204	[OPI-02]	222	[GGAGGAGAGGTAGCGGTCGG]	[TCTCCTCCTTCTTCCCTC]
635) >GT119243	[OPI-02]	204	[GGAGGAGATCTGTTCCAT]	[TCTCCTCCANNNTNNNGG]
636)>GT119507	[OPI-02]	297	[GGAGGAGATTCCGTAAAA]	[CTCTCCTCCTGATCTTTCT]
637) >GT119640	[OPA-03]	236	[AGTCAGCCAAAGTGCGACC]	[TGGCTGACTACAACATCCA]
638)>GT119884	[OPE-04]	252	[GTGACATGGCATTTCATC]	[GCATGTCACTCGCTGGCAT]
639)>GT120373	[OPI-09]	291	[TGGAGAGCGTCTTGTTAG]	[TGCTCTCCATTCTCTTTCG]
640)>GT120387	[OPY-19]	235	[TGAGGGTCTTCACAAAAA]	[GACCCTCACTGGAAAGAC]
641) >HQ385987	[OPI-02]	268	[GGAGGAGAAGAAGAAGAA]	[TCTCCTCCACCTTCATAT]

--
